# Identifying island safe havens to prevent the extinction of the World’s largest lizard from global warming

**DOI:** 10.1002/ece3.6705

**Published:** 2020-09-15

**Authors:** Alice R. Jones, Tim S. Jessop, Achmad Ariefiandy, Barry W. Brook, Stuart C. Brown, Claudio Ciofi, Yunias Jackson Benu, Deni Purwandana, Tamen Sitorus, Tom M. L. Wigley, Damien A. Fordham

**Affiliations:** ^1^ The Environment Institute and School of Biological Sciences The University of Adelaide Adelaide SA Australia; ^2^ Department for Environment and Water Adelaide SA Australia; ^3^ Centre for Integrative Ecology School of Life and Environmental Sciences Deakin University Waurn Ponds Vic. Australia; ^4^ Komodo Survival Program Bali Indonesia; ^5^ School of Natural Sciences University of Tasmania Hobart Tas Australia; ^6^ Department of Biology University of Florence Sesto Fiorentino Italy; ^7^ Komodo National Park Flores Indonesia; ^8^ Balai Besar Konservasi Sumber Daya Alam Kupang Indonesia; ^9^ Climate and Global Dynamics Laboratory National Center for Atmospheric Research Boulder CO USA

**Keywords:** climate change, conservation management, demographic model uncertainty, extinction risk, population viability, sea‐level rise

## Abstract

The Komodo dragon (*Varanus komodoensis*) is an endangered, island‐endemic species with a naturally restricted distribution. Despite this, no previous studies have attempted to predict the effects of climate change on this iconic species. We used extensive Komodo dragon monitoring data, climate, and sea‐level change projections to build spatially explicit demographic models for the Komodo dragon. These models project the species’ future range and abundance under multiple climate change scenarios. We ran over one million model simulations with varying model parameters, enabling us to incorporate uncertainty introduced from three main sources: (a) structure of global climate models, (b) choice of greenhouse gas emission trajectories, and (c) estimates of Komodo dragon demographic parameters. Our models predict a reduction in range‐wide Komodo dragon habitat of 8%–87% by 2050, leading to a decrease in habitat patch occupancy of 25%–97% and declines of 27%–99% in abundance across the species' range. We show that the risk of extirpation on the two largest protected islands in Komodo National Park (Rinca and Komodo) was lower than other island populations, providing important safe havens for Komodo dragons under global warming. Given the severity and rate of the predicted changes to Komodo dragon habitat patch occupancy (a proxy for area of occupancy) and abundance, urgent conservation actions are required to avoid risk of extinction. These should, as a priority, be focused on managing habitat on the islands of Komodo and Rinca, reflecting these islands’ status as important refuges for the species in a warming world. Variability in our model projections highlights the importance of accounting for uncertainties in demographic and environmental parameters, structural assumptions of global climate models, and greenhouse gas emission scenarios when simulating species metapopulation dynamics under climate change.

## Graphical abstract

The Komodo dragon (*Varanus komodoensis*) is an endangered, island‐endemic species with a naturally restricted distribution. Despite this, no previous studies have attempted to predict the effects of climate change on this iconic species. Our modeling shows that without quick action to mitigate climate change impacts on Komodo National Park and on the island of Flores, we risk committing Komodo dragons—a globally iconic species—to extinction.

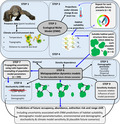

## 1 INTRODUCTION

Tropical island endemics are highly vulnerable to global change (Sodhi, Koh, Brook, & Ng, 2004). Island species often possess attributes, such as low dispersal rates, small population sizes, low heterozygosity, and strong local adaptation, which constrain the ecological and evolutionary responses needed to counter rapid environmental change (Fordham & Brook, 2010; Frankham, 1998). The impact of climate change on tropical island species may be exacerbated because tropical species are particularly susceptible to increases in temperature associated with global warming (Tewksbury, Huey, & Deutsch, 2008) and sea‐level rise is projected to flood low‐lying island habitats, leading to a marked deterioration in quality and availability of habitats (Menon, Soberón, Li, & Peterson, 2010; Wetzel, Kissling, Beissmann, & Penn, 2012). Consequently, the impacts of anthropogenic climate change are expected to be disproportionately large for tropical archipelagos, such as Indonesia, which harbor extremely high levels of endemism (Sodhi et al., 2004).

The Komodo dragon *Varanus komodoensis* is the world's largest lizard and a unique and iconic island‐endemic species. They occupy a highly restricted range distribution (Ciofi & De Boer, 2004) and are considered “Vulnerable” by the International Union for Conservation of Nature (IUCN, 2017). However, the last IUCN Red List assessment for the species was done over 20 years ago (World Conservation Monitoring Centre, 1996), and since this time, there has been considerable research on trends in Komodo dragon population abundance and range area (Imansyah, Jessop, Ciofi, & Akbar, 2008; Jessop et al., 2007, 2018; Purwandana et al., 2014, 2015). Therefore, a reassessment of the present‐day threat status of Komodo dragons is overdue.

**Figure 1:**
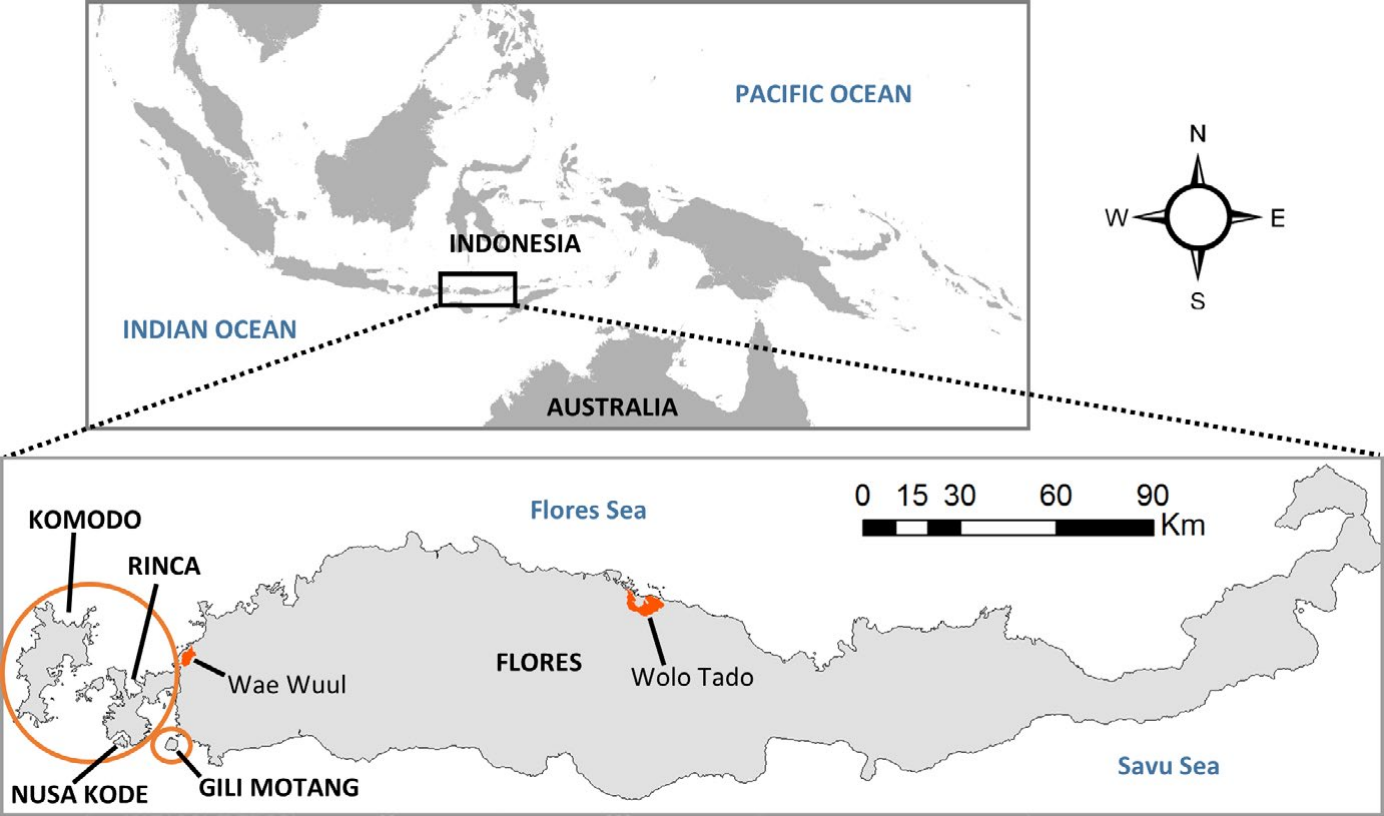
The current‐day geographic range of Komodo dragons is spread across five islands in East Nusa Tenggara, Indonesia. Islands within the Komodo National Park are indicated by orange circles. Orange shading shows the location of reserves on Flores

Populations of Komodo dragons currently persist on five islands in southeastern Indonesia (Figure 1). Fewer than 3,000 individuals live in an area of ~600 km^2^, split across the islands of Komodo, Rinca, Nusa Kode, and Gili Motang within the World Heritage‐listed Komodo National Park (KNP; Purwandana et al., 2014). On the largest island of Flores (13,540 km^2^), outside of KNP’s boundaries, range contraction and population declines have been documented, mainly due to expansion of human settlements, illegal hunting of prey species, and forest clearance for agriculture (Ariefiandy, Purwandana, Natali, et al., 2015; Ciofi & De Boer, 2004). Komodo dragons on Flores are currently only found in a small number of habitat fragments along the north and west coasts of the island. Only ~80 km^2^ of potential Komodo dragon habitat is protected for conservation purposes on Flores (Ariefiandy, Purwandana, Nasu, Surahman, & Jessop, 2015; Ciofi & De Boer, 2004).

Komodo dragons have persisted for millennia in their current range despite interactions with early hominids and substantial climatic‐ and sea‐level‐related habitat changes during the last glacial maxima (36–17 ka; van den Bergh et al., 2009) and subsequent Holocene warming. However, climate change models project that over the next century, Indonesia will experience unprecedented rates of both temperature rise and reduced rainfall (Boer & Faqih, 2004; Diffenbaugh & Giorgi, 2012), leading to a prolonged dry season with increased fire frequency and decreased soil moisture (Fernandes et al., 2017). This is projected to cause a contraction of mesic forest cover and an expansion of drier vegetation communities, such as savannah woodland (Bickford, Howard, Ng, & Sheridan, 2010). This vegetation transformation is likely to negatively impact Komodo dragons by altering habitat and prey availability with impacts on both survival and reproduction (Jessop et al., 2006, 2007). In addition, rising sea levels are likely to inundate the low‐lying valleys (Heaney, 1991) that currently support the highest densities of Komodo dragons, leading to a permanent loss of their preferred lowland habitat (Purwandana et al., 2014). When combined with the existing issues of human‐induced habitat loss, this could be disastrous for the species.

Given the Komodo dragon's potential susceptibility to climate change and its economic importance to Indonesia through tourism (Walpole & Goodwin, 2000; Walpole & Leader‐Williams, 2002), on‐ground resource managers urgently need near‐term projections of Komodo dragon metapopulation dynamics to inform climate change conservation policies. Our specific aims were to: (a) estimate the effects of projected regional temperature increase and sea‐level rise on the habitat suitability, metapopulation structure, and total population size of Komodo dragons between 2010 and 2050, (b) determine whether the risk of extirpation differed across island populations, and (c) investigate the effect of structural uncertainties in global climate models on projections of extinction risk for Komodo dragons.

The novelty of our approach to range dynamics modeling under climate change is the inclusion of multiple sources of uncertainty in a process‐based model, including important structural uncertainties in coupled atmosphere–ocean general circulation models (AOGCMs), which are often ignored in ecological studies. Accounting for AOGCM structural uncertainties in climate change projections is important, because assumptions regarding climate sensitivity (the equilibrium global mean warming for a doubling of CO_2_ concentration) and aerosol forcing can have an equally strong influence on projections of regional climate change as the choice of greenhouse gas emission scenario (Fordham, Wigley, & Brook, 2011; IPCC, 2013). We show that although choice of AOGCM structure causes large uncertainty in ecological model projections of future range and abundance of Komodo dragons, global warming is forecast to cause the extirpation of entire island populations in the near future.

## 2 METHODS

**Figure 2:**
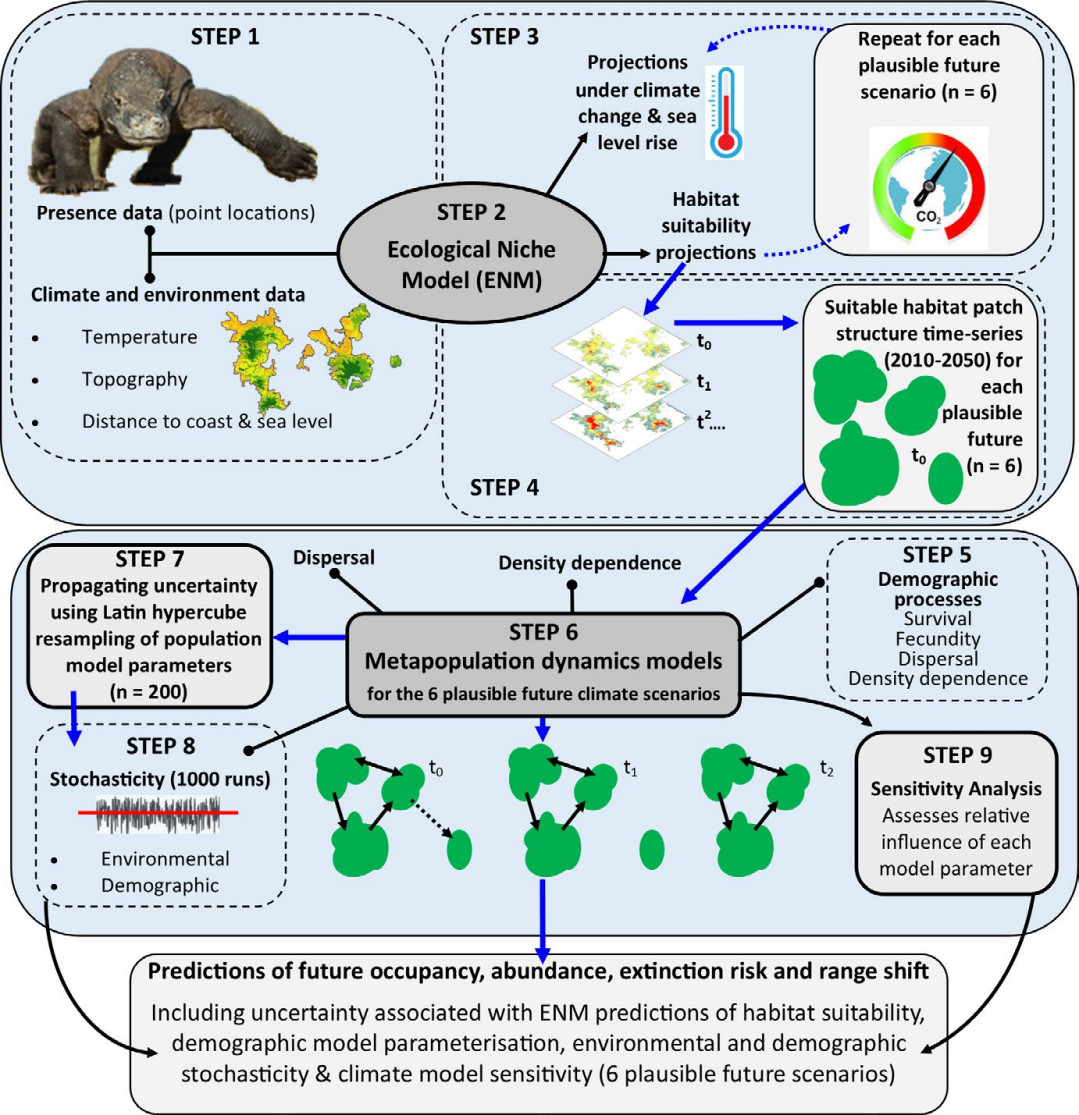
Schematic diagram illustrating our method for coupling ecological niche (ENM) and stochastic population models to create a niche‐population model (NPM), which was used to predict the future range dynamics of Komodo dragons. The presence and environmental data (step 1) were used in the ENM (step 2) to project range‐wide, annual, habitat suitability under current and future climate conditions (step 3). Habitat suitability projections from the ENM (2010–2050) were used to define the spatial habitat patch structure (step 4) for stochastic metapopulation models (step 5) that simulated spatiotemporal abundance and occupancy patterns across all five islands of the species’ global distribution (the NPM, step 6). Simulations accounted for environmental and demographic parameter uncertainty in the NPM (steps 7 and 8), with sensitivity analysis used to identify parameters exerting the greatest influence on the model projections (step 9)

To predict the potential effects of future climate change on Komodo dragons, we used a recently developed approach that couples an ecological niche model (ENM, also known as a species distribution model) and a metapopulation simulation model (spatially explicit population viability analysis), to create a coupled niche‐population model (NPM). The NPM was used to simulate landscape‐scale population processes, including dispersal and source–sink dynamics, across the species’ geographic range, allowing projections of metapopulation abundance, island population extirpation rates, and habitat patch occupancy under various climate change scenarios (Figure 2; Fordham, Akçakaya, Araújo, Keith, & Brook, 2013; Fordham, Mellin, et al., 2013).

The NPM approach has several advantages over alternative methods, such as pattern‐based ENMs or nonspatial population viability analyses. First, it permits integration of dispersal and metapopulation dynamics into forecasts of a species’ geographic range, providing more ecologically realistic predictions of a species’ response to climate change (Anderson et al., 2009). Second, it yields direct estimates of extinction risk, in addition to vulnerability measures based on projected changes in range area, habitat patch occupancy, and total habitat suitability (Fordham, Akçakaya, et al., 2013). Third, this approach inherently incorporates demographic responses to multiple, and often synergistic, processes of global change (Fordham, Resit Akçakaya, et al., 2012). Together, these advantages mean that, where data permit, coupled NPMs provide better predictions of range and population dynamics in response to climate change (Fordham et al., 2018).

Komodo dragons have been the focus of intensive long‐term and large‐scale population monitoring efforts, generating temporal and spatial occupancy and demographic data (Ariefiandy, Purwandana, Natali, et al., 2015; Ciofi et al., 2007; Purwandana et al., 2014, 2015; Sastrawan & Ciofi, 2002). We used these data to parameterize our NPM and make predictions of climate change impacts on the future abundance and distribution of Komodo dragons. We allowed uncertainty in key demographic parameters (such as survival rates, dispersal, and density‐dependant population growth rates), and in ecological niche model projections of habitat suitability, to propagate through to model projections of future Komodo dragon range and abundance (Fordham, Haythorne, & Brook, 2016). We accounted for important structural assumptions in AOGCM climate change projections, as well as greenhouse gas emission scenario, on predictions of extinction risk for Komodo dragons. To do this, we considered six different plausible future climate scenarios from an ensemble of AOGCMs (Fordham et al., 2011). Each scenario was based on different combinations of global greenhouse gas emissions and climate model structural parameters for climate sensitivity to CO_2_ concentration and aerosol forcing. AOGCM sensitivity is classified by the amount of temperature change resulting from a doubling of atmospheric CO_2_ concentration since industrialization (Wigley et al., 2009). Aerosol forcing in AOGCMs accounts for the particulates released by the burning of fossil fuels and biomass, which have a net cooling effect on global temperatures (by blocking sunlight; IPCC, 2013; Wigley & Santer, 2013).

### 2.1 Ecological niche model

We modeled the current distribution of Komodo dragons using geographically referenced occurrence data (*N* = 4,028; see Appendix S1, Table S1.1), intersected with climate and landscape variables at a 1 × 1‐km grid‐cell resolution (see step 1 in Figure 2). We built a consensus ensemble ecological niche model (ENM; step 2 in Figure 2) using the R package biomod2 (Marmion, Parviainen, Luoto, Heikkinen, & Thuiller, 2009; Thuiller, Lafourcade, Engler, & Araújo, 2009). We used four different modeling algorithms (generalized linear models, generalized additive models, generalized boosted‐regression models, and MAXENT) with interactions allowed, but depth limited to 1 in the GLM and GAM models, and 3 in the GBM models. We used an ensemble ENM approach because it avoids potentially important biases inherent with using single ENM models (Araújo et al., 2019; Araújo & New, 2007). We weighted the contribution of each of the individual ENMs in the ensemble using the True Skill Statistic (TSS) model evaluation scores. In doing so, individual models that had better evaluation scores contributed more to the ensemble model output (Marmion et al., 2009).

Our correlative ENM approach is based on the fundamental assumption that the distribution of a species, in approximate equilibrium with its environment, is a good indicator of its ecological requirements (Elith & Leathwick, 2009). Use of proximal predictors of ecological suitability (i.e., those that directly influence geographic distributions or demographic mechanisms) is the gold standard for reducing uncertainty in projections from species distribution models (Araújo et al., 2019). However, distal predictors, including landscape and some climate variables, can be used as proxies for proximal constraints such as habitat and prey availability (Elith & Leathwick, 2009). Because robust spatiotemporal data were not available to generate some proximal predictors of Komodo dragon geographic range (e.g., vegetation community and prey abundance), we used available distal surrogates (Appendix S1). In this context, temperature can be considered both a proximal and distal predictor variable, in that it has both a direct (proximal) influence on Komodo dragon physiology (and therefore fitness) and behavior, and an indirect (distal) influence on vegetation (habitat) and prey distributions.

We evaluated the ENM using both independent evaluation (by means of a separate dataset for Komodo dragon occupancy collected with camera traps) and cross‐validation. The independent data provide a more stringent test of the predictive ability of the ENM for the current day (Elith & Leathwick, 2009). The calibration and evaluation of the ENM are described in detail in Appendix S1. The ensemble ENM was used to project future Komodo dragon habitat suitability at annual time steps (up to 2100) under AOGCM‐projected changes in temperature and sea level for the six “plausible future” scenarios (step 3 in Figure 2). The resulting ENM projections of habitat suitability under the six “plausible futures,” when taken together, accounted for uncertainties in greenhouse gas emission policy and AOGCM structural assumptions (see 2.1.2).

The six plausible future time series of Komodo dragon habitat suitability generated by the ensemble ENM were then used as a proxy for upper abundance (carrying capacity, *K*) in the coupled NPM (VanDerWal, Shoo, Johnson, & Williams, 2009; see step 4 in Figure 2). To relate ENM model projections of habitat suitability to *K*, we used an iterative approach to optimize the relationship between these two variables based on target values of current abundance (after Anderson et al., 2009) for each island taken from previously published studies (see Appendix S1). We only projected up to 2050 so that we avoided extrapolating to novel climates (Elith, Kearney, & Phillips, 2010; Elith & Leathwick, 2009) where mean temperatures after 2050 exceeded those of the ENM training dataset. This approach, while widely used as a method for avoiding extrapolation, assumes that the covariance structure of predictors remains constant through time. However, covariance structures can, in some instances, change through time (Jackson, Betancourt, Booth, & Gray, 2009).

#### 2.1.1 Current climate and environmental spatial data

Through a literature review and consulting with species experts (including authors on this paper), we identified temperature, topography, and distance from the coast as the landscape and climate variables likely to have the greatest impact on Komodo dragon distribution either directly or indirectly (by, e.g., influencing the distribution of their prey species).

Temperature directly affects the physiology of Komodo dragons, limiting occurrence in cooler, higher (above ~500 m) areas, as well as the hottest lowland areas (Auffenberg, 1981), and is also likely to indirectly affect distribution via its impact on vegetation communities and prey species distributions. Distance from the coast and slope are good proxies for land/vegetation cover (and therefore prey availability) because higher and steeper areas drive orographic rainfall patterns, which in turn affect vegetation type. Topographic slope can also act as a physical barrier to Komodo dragon movement. Higher altitudes (>600 m elevation) and moist forest habitats constrain the distribution of the Komodo dragon, causing them to rarely be found more than 5–6 km from the shoreline (Auffenberg, 1981). Conversely, flatter and drier areas are commonly characterized by lowland savannah woodland and lowland deciduous forests (<300 m elevation), the latter being the most preferred habitat for Komodo dragons (Auffenberg, 1981) and their prey (Purwandana et al., 2015).

We modeled temperature as a function of elevation, based on the moist adiabatic lapse rate. We first calculated a long‐term sea‐level average temperature baseline of 26.38°C (*n* = 56 years of observational data). We then applied an elevation lapse rate of 6.1°C decline per 1,000 m increase in altitude (Harris et al., 2014; Raxworthy et al., 2008) using a 250‐m horizontal resolution digital elevation model (Shuttle Radar Topography Mission [SRTM]; Jarvis, Reuter, Nelson, & Guevara, 2008). We then upscaled the temperature raster to 1‐km grid‐cell resolution by multicell averaging. Further methodological details are available in Appendix S1. We used mean rather than minimum or maximum temperature in our models because there is low diurnal and seasonal variance in temperature in our study region (due to its equatorial location) and because long‐term mean temperature was the metric most reliably recorded by the few weather stations located in the region.

Topographic slope was calculated as the maximum rate of change in elevation from each cell to all eight neighboring cells using the SRTM elevation raster and the slope tool in ArcGIS (v 10.3.1). We upscaled slope from the original SRTM cell size (250 m) to the 1‐km grid‐cell resolution by summing the number of smaller constituent cells (*n* = 16) that had a slope value greater than the 75th percentile for slope across the entire study area. Higher values in the final slope raster, therefore, indicate that a greater proportion of the cell area consisted of steep slopes.

We calculated distance from the nearest coastline to each grid cell in the study region using the 1 × 1‐km resolution elevation raster. Cells with a mean elevation of <0 m were classified as the sea, while cells with mean elevation >0 m were assigned to land, enabling us to define the island's coastlines at the boundary between land and sea cells. We then calculated the distance from the centroid of each cell to the nearest point on the coastline using the R package “sp” (Bivand, Pebesma, & Gomez‐Rubio, 2013). The distance to coast value for each grid cell was recalculated at each time step to account for sea‐level rise (see details in section 2.1.22).

Our choice of spatial predictors was influenced, to some extent, by the availability of additional robust predictors of climate and environmental conditions. For example, commonly used estimates of rainfall and temperature, those based on the interpolation of meteorological data (i.e., downloadable from WorldClim2), were investigated and found to be highly uncertain for our study region (Fick & Hijmans, 2017; specifically their Figure 4). This is likely due to the complex topography and the sparseness of weather stations in the region. We explored the relationship between WorldClim 2 projections for rainfall and temperature and found them to be strongly correlated. The correlations for these datasets in our study area were between −0.73 and −0.94 (depending on the specific area of interest), indicating that as temperature increases, precipitation decreases. This relationship is likely to be due to the approach used to spatially interpolate the climate data, whereby a splining algorithm that uses elevation (which is highly correlated with temperature) as a covariate is used to spatially project precipitation (Fick & Hijmans, 2017). These exploratory analyses are shown in Appendix S1 (Table S1.2) and provide strong justification for not including precipitation as a predictor variable in an SDM that already contains temperature (Dormann et al., 2013). Furthermore, some of the ENM algorithms we applied in biomod2 perform poorly with colinear predictors (Dormann et al., 2012).

We explored the utility of several land use and land cover layers, but were unable to find one that reliably separated suitable and unsuitable land cover classes for Komodo dragons (these exploratory investigations are discussed in detail in Appendix S1, Figures S1.1 and S1.2). In addition, there were no datasets available that included future predicted land use/land‐use change for our study region. These are required for generating projections of habitat suitability from the ENM, unless the current spatial pattern in land use is assumed to remain static over time, which is unrealistic (Hof et al., 2018). The implications of not including land‐use data in this study are addressed in the Discussion.

#### 2.1.2 Projected changes in temperature and sea level

We considered six different plausible future climate scenarios using differing projections from an ensemble of climate models run under two contrasting global greenhouse gas emission scenarios from the RCP set (van Vuuren et al., 2011): one “business as usual” (reference, or *Ref*) scenario (RCP 8.5) and one stringent emission mitigation (policy, or *Pol*) scenario (RCP 2.6). These two emission scenarios were chosen because they span the extremes of the range of scenarios generally considered, and so encompass the likely range of potential outcomes from climate change (van Vuuren et al., 2011). We further parameterized the *Ref* and *Pol* emission scenarios described above with three differing climate sensitivities to CO_2_ concentration and aerosol forcing (*Low*,* Mid,* and *High*). This resulted in six different sets of future climate projections (the six “plausible future climate scenarios,” Table 1), which account for uncertainty in the structural assumptions associated with AOGCMs.

**Table 1:** Details of the six plausible future scenario climate model parameterizations

Future climate scenario	Emission scenario	Sensitivity (ΔT2x)	Aerosol forcing
Pol Low	A very low greenhouse gas emission scenario, assuming considerable intervention. Based on IPCC Representative Concentration Pathway (RCP) 2.6: Climate policies lead to greenhouse gas emissions peaking between 2010 and 2020 and declining thereafter.	ΔT2x 5th percentile = 1.5°C	Low (−0.3 W/m^2^) = 5th percentile based on the uncertainty around the “best estimate” value given in the 2007 IPCC report
Pol Mid		ΔT2x 50th percentile = 3°C	Mid (−0.7 W/m^2^) = the median (best estimate) for aerosol forcing given in the 2007 IPCC report
Pol High		ΔT2x 95th percentile = 6°C	High (−1.1 W/m^2^) = 95th percentile based on the uncertainty around the “best estimate” value given in the 2007 IPCC report
Ref Low	A high greenhouse gas emission scenario with a rising radiative forcing pathway. Based on IPCC Representative Concentration Pathway (RCP) 8.5: Lack of climate policies leads to greenhouse gas emissions continuing to rise throughout the 21st Century.	See Policy low	See Pol low
Ref Mid		See Policy mid	See Pol mid
Ref High		See Policy high	See Pol high

Note

Model sensitivity to CO_2_ concentration is classified by the amount of temperature change resulting from a doubling in atmospheric CO_2_ concentration (ΔT2x). Aerosol forcing accounts for the particulates released by the burning of fossil fuel and biomass, which have a net cooling effect on global temperatures (by blocking sunlight).

We used an ensemble of seven atmosphere–ocean general circulation models (AOGCMs) to generate robust projections of regional changes in temperature, for each of the six plausible futures, on a 2.5‐by‐2.5‐degree grid. The ensemble included a mixture of globally (GFDLCM20, MIROCMED, MRI‐232A, UKHADCM3) and regionally (BCCRBCM2, CCSM‐30, CSIRO‐30) skillful AOGCMs (Harris et al., 2014). Skill was assessed based on the ability of the AOGCM to replicate observed climatic conditions (Fordham et al., 2011). Future temperature and sea‐level change time series were generated for three (2.5° × 2.5°) grid cells delineating the study region. For each of the six plausible future climate scenarios (Table 1), we generated multimodel‐averaged AOGCM projections for change in mean temperature across the study region and mean global sea level (from a 20‐year baseline centered on the year 2000) at 5‐year time steps for the period 2010–2100 using MAGICC/SCENGEN version v5.3.2 (Fordham, Wigley, Watts, & Brook, 2012) and MAGICC sea level model v2.0 (Nauels, Meinshausen, Mengel, Lorbacher, & Wigley, 2017).

The five‐year projections of temperature and sea‐level change were linearly interpolated to annual time steps and then downscaled to match the spatial resolution of the landscape variables (1‐km grid cells) using the “change factor” empirical method, whereby the low‐resolution change from a AOGCM is added directly to a high‐resolution baseline of observed climatology (Hulme, Raper, & Wigley, 1995). Similarly, we applied the annual sea‐level forecasts to the land elevation raster by subtracting the projected amount of sea‐level rise from the elevation value at each 1‐km grid cell. Thus, the future spatial layers of distance from coast explicitly take account of forecast sea‐level rise by removing grid cells that were projected to become permanently inundated (under a simple "bath‐tub" sea‐level rise approach, e.g., Traill, Bradshaw, Delean, & Brook, 2010) and recalculating distance to coast for remaining “land” areas for each year up to 2050. AOGCM ensemble‐averaged annual projections of temperature and sea‐level rise across the distribution of Komodo dragons for each of the six plausible futures are shown in Appendix S2 (Figures S2.1 and S2.2).

### 2.2 Capture–mark–recapture analyses

We developed capture–mark–recapture (CMR) models in “Rmark” (Laake, 2013) to estimate annual survival rates for each age class of Komodo dragons (hatchlings, juveniles, and adults), across the different island populations (step 5 in Figure 2). These analyses used data collected from more than 1,000 marked individuals over six capture occasions between 2003 and 2013 (Purwandana et al., 2014, 2015). Komodo dragons from the small islands within KNP (Figure 1: Gili Motang and Nusa Kode) grow slower and mature at a smaller size than those on the large islands in KNP (Figure 1: Komodo and Rinca; Laver et al., 2012). Age class thresholds were adjusted to account for this interisland variation in age and size of maturity (Appendix S1, Table S1.3). The CMR survival analysis is described in detail in Appendix S1.

### 2.3 Coupled niche‐population model (NPM)

We simulated the metapopulation dynamics of Komodo dragons across the species’ entire current‐day range using stage‐based stochastic matrix models in RAMAS GIS v5 (Akcakaya & Root, 2005) and a coupled niche‐population modeling (NPM) framework (Fordham, Akçakaya, et al., 2013). We used these models to project changes in range‐wide and island‐specific abundance, patch occupancy, and risk of extinction under the six plausible future climate scenarios (step 6 in Figure 2). Our NPM framework assumes that estimates of apparent survival rates (from CMR models of different island populations) capture responses to varying environmental conditions, such as prey availability, which is known to vary between islands (Ariefiandy et al., 2016; Ariefiandy, Purwandana, Coulson, Forsyth, & Jessop, 2013; Jessop et al., 2007; Laver et al., 2012; Purwandana et al., 2014, 2015, 2016).

The spatial structure of the modeled metapopulation (the size and location of subpopulations within and across island populations) was based on annual predictions of habitat suitability from the ENM (step 4 in Figure 2) transformed to carrying capacity (*K*). The transformation used a formula that was optimized iteratively to ensure that the baseline abundance on each island approximated recent island‐based upper abundance estimates (see Appendix S1, Table S1.4; Jessop et al., 2007; Purwandana et al., 2014; Purwandana et al., 2015). This optimization approach is widely used when configuring NPMs (Anderson et al., 2009) and addresses the problem that the relationship between ENM suitability and upper abundance is not necessarily linear (Thuiller et al., 2014). We applied a multiplier to carrying capacity for habitat within large protected areas. This was based on work by Purwandana et al. (2015), which assessed habitat quality and prey availability in different parts of the species’ range. The effect of this decision on model results was tested using a sensitivity analysis. We accounted for uncertainty in the association between ENM projections of Komodo dragon habitat suitability and upper abundance by simulating *K* within uncertainty bounds, allowing this uncertainty to propagate through to the model results (see section 2.3.11).

We used a cell clustering algorithm to join all cells with habitat suitability values greater than a set threshold value (0.6) and within a neighborhood distance (1.3 km) into habitat patches. These were then treated as panmictic populations in the model (Akcakaya & Root, 2005). The minimum habitat suitability threshold used in the algorithm was determined iteratively to optimize model estimates of current‐day habitat patch number and distribution and extant population size (Anderson et al., 2009). The estimate of neighborhood distance (1.3 km) was based on Komodo dragon home range and movement studies from Komodo National Park (Ciofi et al., 2007; Jessop et al., 2018; Sastrawan & Ciofi, 2002; Appendix S1, Figure S1.3). A detailed description of the approach used to delineate the spatial structure of the metapopulation is in Appendix S1. The entire Komodo dragon metapopulation for the present day, thus consisted of 43 panmictic populations, spread across five islands.

We assumed that dispersal between islands does not occur based on recent genetic, CMR, and radiotelemetry evidence (Jessop et al., 2018). Density dependence was modeled for all populations using a Ricker logistic equation, which assumed worsening returns as the populations of juvenile and adult Komodo dragons increase and the amount of resources per individual decreases (Akcakaya & Root, 2005). This parameterization was supported by a previous study showing that prey availability limited Komodo dragon population size, indicating strong competition for resources (Purwandana et al., 2015). Hatchlings were excluded from the density‐dependent calculation because they are arboreal and are therefore not competing for the same resources as the older life stages. A detailed description of the population model parameterization is given in Appendix S1.

Demographic processes were simulated using a stage‐structured, postbreeding, female‐only stochastic population model (step 6 in Figure 2). We modeled only females because there is no strong evidence that vital rates differ between sexes and the species is polygamous (Purwandana et al., 2014, 2015). The stochastic models were parameterized using stage class‐specific survival rates (see CMR analysis), fecundity rates, and dispersal rates. The maximum rate of intrinsic population growth (Rmax) for all populations was estimated to be 1.18 based on our own Pradel models of population growth and previous studies by Purwandana et al. (2014). We accounted for interisland differences in survival rates based on our CMR estimates (see Appendix S1 for details).

#### 2.3.1 Simulations

We ran six baseline spatiotemporally explicit NPMs, one for each of the six plausible future climate scenarios (described in Table 1 and step 6 in Figure 2). This allowed us to assess the effect of structural uncertainties in AOGCMs and the choice of greenhouse gas emission scenario on the range dynamics of Komodo dragons and their extinction risk from climate change. The demographic parameters of these six baseline NPMs, and the ENM‐derived predictions of *K,* were varied (within uncertainty bounds) using Latin hypercube sampling (*n* = 200) and the Sensitivity Analysis of Range Dynamics Model (SARDM) software (Fordham et al., 2016; see step 7 in Figure 2 and Appendix S1, Table S1.5). We varied these parameter values to account for documented and suspected uncertainties in demographic (e.g., vital rates, density dependence, Rmax) and environmental parameters (e.g., uncertainty in the association between projected habitat suitability and upper abundance). Each NPM was run at annual time steps from 2010 to 2050, with a 20‐year burn‐in period, using 2010 environmental conditions to achieve an equilibrium metapopulation structure (step 8 in Figure 2). Each run of the NPM included 1,000 simulations that accounted for environmental and demographic stochasticity (which resulted in a total of 1,200,000 NPM iterations: 200 Latin hypercube iterations of the six plausible future NPMs, each run 1,000 times with stochasticity).

#### 2.3.2 Niche‐Population Model (NPM) output

Under each climate change scenario, we report the median (50th percentile of all model simulations) number of female Komodo dragons projected to be alive each year (2010 to 2050), and the annual relative change in habitat patch occupancy to 2050 (bounded by the 5th and 95th percentiles from all simulations) for the entire metapopulation and for each island separately. We also report the median expected minimum abundance (EMA), which is a continuous integrated metric reflecting risks of both a decline in abundance and extinction risk (McCarthy & Thompson, 2001). In addition to these outputs, we also report the results of a sensitivity analysis using SARDM, which was done for two greenhouse gas emission scenarios (RCP 8.5 and RCP 2.6), both with midrange climate sensitivity and aerosol forcing (step 9 in Figure 2).

## 3 RESULTS

### 3.1 Ecological niche model (ENM)

Evaluation of the ensemble ENM showed that the model had good predictive skill based on cross‐validation, with True Skill Statistic (TSS) and area under the curve (AUC) scores of 0.583 and 0.842, respectively (Allouche, Tsoar, & Kadmon, 2006; Swets, 1988). Independent validation of our ensemble ENM confirmed a good level of predictive skill (Boyce Index = 0.915). Together, these tests suggest good evaluation skill. Furthermore, the model independently predicted Komodo dragon presences on Flores Island at the few known sites of current‐day occurrence. These are the western end of the island around the Wae Wuul Reserve and in a strip along the central‐northern coast of the island from Pota in the west to the east of Riung including the Wolo Tado Reserve (Ariefiandy, Purwandana, Nasu, et al., 2015; Ariefiandy, Purwandana, Natali, et al., 2015; Ciofi & Gibson, 2006; Forth, 2010). This verification provides some confidence in the transferability of the model in space.

Random permutations of predictor variables showed that temperature and distance from the coast were the most important predictors of habitat suitability, with topographic slope contributing little additional explanatory power (relative variable importance weights were 0.457, 0.504, and 0.039, respectively). The models indicated that warmer areas (but not the hottest), which are located close to the coast, contained the most suitable habitat for Komodo dragons (Appendix S3, Figure S3.1).

**Figure 3:**
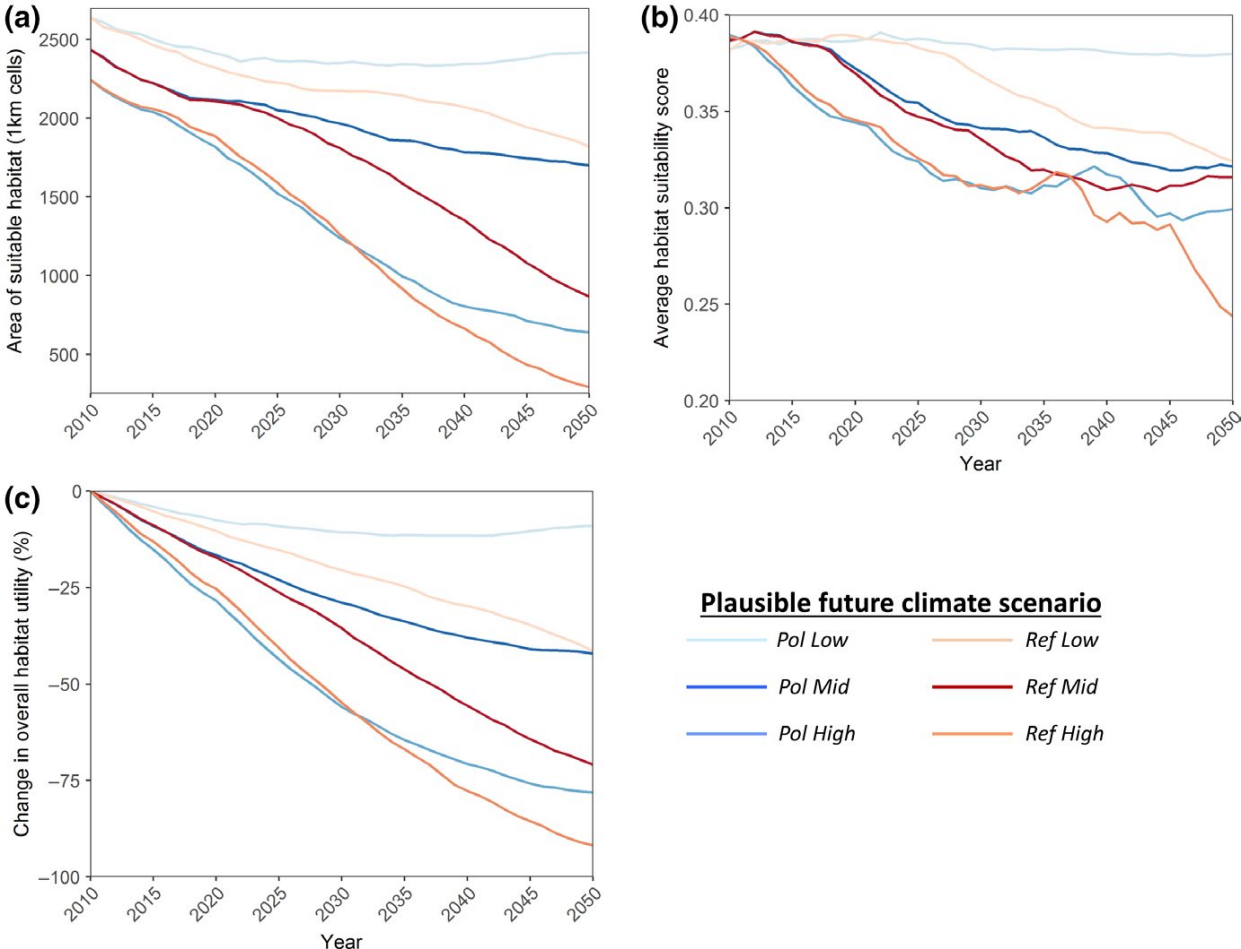
Projections of habitat metrics from ecological niche models (ENM) for the global range of the Komodo dragon. ENMs were parameterized with future climate and sea‐level rise data under six plausible future climate scenarios based on varying greenhouse gas emission scenarios (*Ref* [RCP 8.5] and *Pol* [RCP 2.6]), climate sensitivity, and aerosol forcing (*Low*,* Mid,* and *High*). See methods, Table 1 and Appendix S1 for further details. (a) Total area of suitable habitat, (b) average habitat suitability score (i.e., habitat quality), and (c) percentage change in overall habitat utility (a function of both change in habitat suitability and available area). Comparisons of *Ref* and *Pol* scenarios can be made for each climate model sensitivity parameterization (i.e., *Low*,* Mid,* and *High*) to infer the effect of global greenhouse gas emission policy on the projections from the ENM

Projections of future habitat suitability from the ENM for the period 2010–2050 for each of the six plausible future climate scenarios (Table 1) show that large areas of currently suitable habitat are likely to be lost in the future with >1900 km^2^ net loss (>80% of the current range area) under *Ref High*, the most extreme and pessimistic greenhouse gas emission scenario (RCP 8.5) combined with high climate sensitivity and high aerosol forcing. The models predict a concurrent reduction in average habitat suitability under all climate model scenarios except *Pol Low*, the most optimistic future climate scenario, which includes stringent greenhouse gas mitigation (RCP 2.6), combined with low climate sensitivity and low aerosol forcing (see Figure 3a,b, Appendix S3, Table S3.1). Our ENM also showed a reduction in overall habitat utility (calculated by multiplying the average habitat suitability by the area of available habitat at each time step) under all climate scenarios, with estimated decreases of 42% and 71% for the *Pol Mid* and *Ref Mid* scenarios, respectively (Figure 3c). Maps of ENM habitat suitability projections for the Pol Mid and Ref Mid scenarios in 2010, 2030, and 2050 are provided in Appendix S3 (Figure S3.2).

By independently varying future temperature and sea‐level rise in the ENM (while holding the other variables constant), increased temperature was revealed as the primary driver of the reduction in habitat suitability and area (Appendix S3, Figure S3.3). ENM projections through to 2100 indicate an increasing impact of sea‐level rise on habitat suitability beyond 2050 (Appendix S3, Figures S3.3 and S3.4).

### 3.2 Capture–mark–recapture analyses

Annual survival rates differed across the four island populations within KNP and between age classes. Adults on the smaller islands of Kode and Motang were estimated to have lower apparent survival rates (Kode = 0.51, *SE* = 0.16 and Motang = 0.47, *SE* = 0.15, respectively) than juveniles (Kode = 0.62, *SE* = 0.14; Motang = 0.59, *SE* = 0.12). On the larger islands, there was little difference between adult and juvenile apparent survival rates. These were estimated at 0.76 (*SE* = 0.07) and 0.74 (*SE* = 0.08) for adults and juveniles, respectively, on Komodo, whereas on Rinca the estimates were higher, at 0.84 (*SE* = 0.03) and 0.85 (*SE* = 0.03), for adults and juveniles, respectively. The estimated survival rate for hatchlings on Rinca island was 0.2 (*SE* = 0.29).

### 3.3 Coupled niche‐population model (NPM)

**Figure 4:**
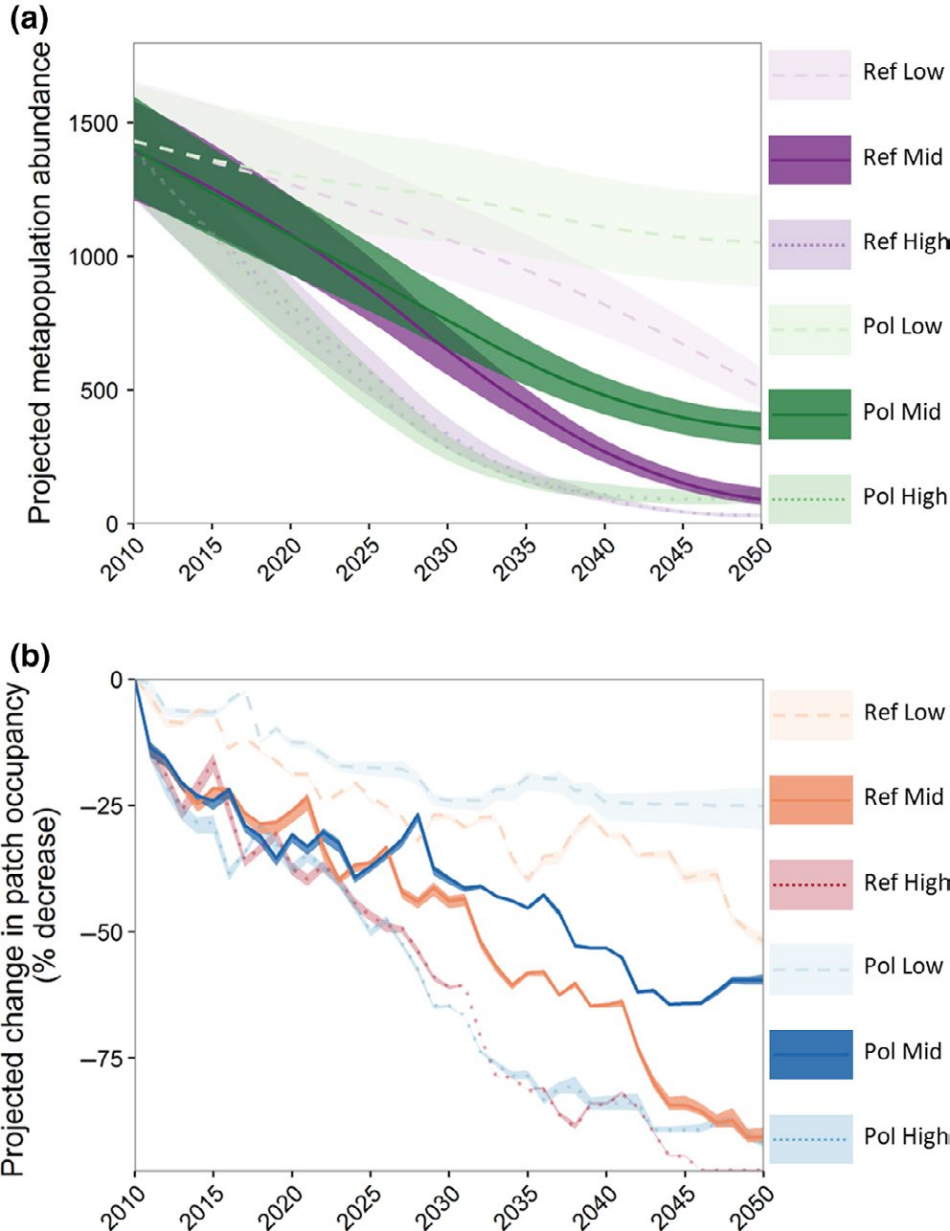
Projected abundance and occupancy for the global distribution of Komodo dragons under six climate change scenarios from 2010 to 2050: (a) abundance of female animals and (b) habitat patch occupancy shown as% decline compared to 2010 levels. Lines indicate the 50th percentile value from 200 coupled niche‐population models (NPM) with plausible demographic parameters (each simulated 1,000 times); shading shows the bounds of the 5th and 95th percentiles. The difference between *Pol* (RCP 2.6) and *Ref* (RCP 8.5) emission scenarios under each of the three climate sensitivity parameterizations (*High*,* Mid,* and *Low*) are indicative of the effect of emission policy on NPM projections

Our NPM predicted a decline in (female) Komodo dragon metapopulation size by 2050 under all six plausible future climate change scenarios (Figure 4). Global greenhouse gas emission scenario (i.e., *Ref*: RCP 8.5; or *Pol*: RCP 2.6) had a strong influence on the simulated trajectories of range‐wide Komodo dragon abundance and patch occupancy. Decreases of >50% were projected for all *Ref* scenarios, compared to >25% for all *Pol* scenarios (under all three climate sensitivity and aerosol forcing scenarios: *Low*,* Mid,* and *High*; Table 2). Future climate scenarios that included global emission mitigation policies (based on RCP 2.6) always resulted in better outcomes for the Komodo dragon, irrespective of the climate sensitivity and aerosol forcing parameterization. However, the size of this “emissions policy effect” decreased as climate sensitivity and aerosol forcing increased (see “benefit of emissions policy” section in Table 2).

**Table 2:** Projected changes in the Komodo dragon metapopulation by 2050 under six plausible future climate scenarios

Future climate scenario	Emission scenario	Aerosol forcing (W/m^2^)	Climate sensitivity (ΔT2x)	Δ abundance	Δ patch occupancy	EMA	Final Occupancy	Final abundance
Ref Low	RCP 8.5	−0.3	1.5°C	−65 (−69, −59)	−52 (−56, −49)	501	20	510
Ref Mid	RCP 8.5	−0.7	3°C	−93 (−94, −89)	−91 (−92, −88)	96	4	103
Ref High	RCP 8.5	−1.1	6°C	−99 (−99, −98)	−97 (−98, −97)	22	1.1	23
Pol Low	RCP 2.6	−0.3	1.5°C	−27 (−39, −15)	−25 (−32, −18)	816	30.4	1,062
Pol Mid	RCP 2.6	−0.7	3°C	−76 (−80, −71)	−60 (−62, −57)	336	17.3	357
Pol High	RCP 2.6	−1.1	6°C	−95 (−96, −95)	−92 (−93, −91)	66	3.2	67

Benefit of emission policy				Abundance	Patch occupancy	EMA	Final Occupancy	Final abundance
Pol Low compared to Ref Low				+38	+27	+315	+10.4	+552
Pol Mid compared to Ref Mid				+17	+31	+240	+13.3	+254
Pol High compared to Ref High				+4	+5	+44	+2.1	+44

Note

Change (Δ) in abundance and habitat patch occupancy (2010 to 2050) are based on the median (50th percentile) from 200 stochastic model simulations (each iterated 1,000 times), with lower/upper confidence intervals indicated by the 5th and 95th percentiles. Expected minimum abundance (EMA), final patch habitat patch occupancy, and final abundance values are shown as the median values (50th percentiles from all model simulations). See Methods for details. The projected benefits of stringent greenhouse gas emission policies on Komodo dragon populations were estimated by calculating the difference between median *Ref* (RCP 8.5) and *Pol* (RCP 2.6) climate model scenarios with the same aerosol forcing and climate sensitivity parameterization.

Niche‐population model projected abundance and occupancy in 2050 decreased with increasing climate model sensitivity and aerosol forcing (Figure 4). Under the most optimistic climate scenario (*Pol Low*), range‐wide metapopulation abundance decreased by 15%–45% by 2050, resulting in an estimated expected minimum abundance (EMA) of 816 females (median value for EMA from 200 plausible NPM models, each run 1,000 times). Under the most pessimistic climate scenario (*Ref High*), the range‐wide metapopulation abundance decreased by 95%–99% by 2050, resulting in an EMA of 22 females. In the absence of a substantial global effort to reduce greenhouse gas emissions, the “most likely” future climate scenario (of the suite that we have tested) is *Ref mid*, which is likely to result in an 89%–94% decrease in range‐wide metapopulation abundance, 88%–92% in habitat patch occupancy, and an EMA of 96 females by 2050. The island‐specific results indicate that most of the remaining individuals in 2050 will be located on Komodo Island (Appendix S4: Table S4.1).

Komodo dragons on the larger protected islands in KNP (Komodo and Rinca) have a higher chance of persisting through to 2050 than those on the smaller protected islands (Motang and Kode) or the largest (but less protected) island of Flores (Appendix S4: Table S4.1). Although considerable declines in both abundance and occupancy are projected for the two larger island populations in KNP (Komodo and Rinca), these populations are likely to persist beyond 2050 under all of the six plausible future climate scenarios we tested, with the exception of Komodo dragons on Rinca under the most extreme plausible future scenario (*Ref High*).

A sensitivity analysis for the NPM shows that the model projections of expected minimum abundance (EMA) were most sensitive to assumptions regarding Rmax (the intrinsic maximum rate of population growth). The next most influential parameter was carrying capacity (*K*; based on ENM predictions of habitat suitability and its relationship to field estimates of upper abundance). The standardized regression coefficients for Rmax and carrying capacity were 0.60 and 0.39, respectively, for the Ref Mid climate scenario (0.99 combined weight), and 0.63 and 0.3, respectively (0.93 combined weight), for the Pol Mid scenario (Appendix S4, Figure S4.1). Changes in these two parameters together had the largest influence on model results for EMA, with higher values of Rmax and carrying capacity resulting in a greater estimate of EMA. These two parameters were consistently the most influential parameters irrespective of the choice of emission scenario (Ref, RCP 8.5; or Pol, RCP 2.6).

Results from a sensitivity analysis that tested the influence of not applying the habitat quality multiplier to small island populations showed that even if we had treated all reserves as equal, the fate of the smaller island populations on Kode and Motang would have remained bleak. There was no difference in predicted extirpations under the reference scenarios and a difference in EMA of just 2–3 individuals under the policy scenarios when the multiplier was applied to these populations (Appendix S4, Table S4.2).

## 4 DISCUSSION

The aims of our study were to estimate the effects of projected regional temperature and sea‐level increases on Komodo dragon habitats and populations, as well as to understand how important structural uncertainties in climate model projections can affect projections from ecological models. We show that climate change over the next few decades will likely have a major impact on Komodo dragon range and abundance, potentially causing the extirpation of whole island populations and ultimately threatening the viability of the species in the near future.

Our results show an overall decline in abundance and habitat patch occupancy for Komodo dragons on all islands, irrespective of the future climate scenario or demographic parameterization of the models. However, the magnitude of the effects of climate change is strongly dependent on both the greenhouse gas emission scenario used (i.e., *Ref* [RCP 8.5] or *Pol* [RCP 2.6]) and key differences in climate model structural assumptions regarding sensitivity and aerosol forcing. The most benign scenario (*Pol Low*) combines low climate model sensitivity to CO_2_ and aerosol forcing with a low greenhouse gas emission scenario. The parameterization of NPMs with this future scenario results in relatively small downward changes in the range and abundance of Komodo dragons compared to other plausible future scenarios. The most severe NPM projections of demographic change was for the *Ref High* scenario (simulated on the basis of high climate sensitivity and aerosol forcing, and under a high global greenhouse gas emission scenario), which resulted in the near range‐wide extinction of Komodo dragons by 2050. Based on the median (most likely) estimates for climate sensitivity and aerosol forcing (the *Mid* scenarios), our models estimate that increased global temperature and sea‐level rise will likely lead to major losses in occupied range area for Komodo dragons by 2050 (57%–92%) and an associated decrease in range‐wide abundance of 71%–94%.

The fate of island populations of Komodo dragon in Komodo National Park (KNP) is also highly dependent on the global greenhouse gas emission scenario used to predict habitat suitability and patch structure into the future (i.e., *Ref*: RCP 8.5; or *Pol*: RCP 2.6). This is, to some extent, a positive result because it means that policies to reduce greenhouse gas emissions have the potential to reduce the negative impact of climate change on Komodo dragons within KNP (Warren, Price, Graham, Forstenhaeusler, & VanDerWal, 2018). Under the *Ref Mid* future climate scenario, populations of Komodo dragons are predicted to persist until 2050 only on the two largest islands in KNP (Komodo and Rinca), while under the *Pol Mid* scenario, all KNP islands are projected to support populations of Komodo dragons to 2050, albeit with reduced abundances. Substantial sea‐level‐related impacts on the range and abundance of Komodo dragons are not predicted until after 2050 (i.e., beyond our NPM projection period; see Appendix S3: Figures S3.3 and S3.4), but they will likely lead to further climate change‐related reductions in Komodo dragon habitat area and habitat quality by the end of the 21st century. Our models predict that Komodo dragons on Flores will be extirpated under all six plausible future climate scenarios in the absence of further conservation management intervention.

To interpret our NPM predictions of near‐future climate change impacts on Komodo dragons, we first need to consider how current environmental conditions influence their distribution. Our ENM identified two key environmental variables to be dominant drivers of Komodo dragon occurrence: temperature and distance from coast. The ENM predicted that areas with moderately warm (25–26°C) mean annual temperatures have a higher probability of presence (i.e., greater habitat suitability) for Komodo dragons compared to areas with either hotter (>26.5°C) or cooler (<24.5°C) mean annual temperatures. Distance from coast had a strong negative influence on modeled habitat suitability for Komodo dragons, with the probability of occurrence decreasing rapidly beyond 2 km from the coastline, owing to a nonlinear interaction between temperature and distance from the coast. This relationship between temperature and distance from coast is likely to strongly influence the distribution and composition of vegetation communities in the study region (Auffenberg, 1981), influencing habitat suitability for Komodo dragons. These ENM‐based inferences are supported by behavioral field observations, where Komodo dragons are generally not observed to be active in open coastal habitats (e.g., savannah grassland) that incur the hottest temperatures, nor in the densely forested, steeper, and higher elevation areas further inland. In contrast, Komodo dragons are more commonly observed in the intermediate areas between these two habitats, which generally contain lowland deciduous forests that grow in a narrow band, extending a maximum of ~5 km inland from the coast (Auffenberg, 1980, 1981; Forth, 2010).

Temperature and distance from coast are also expected to propagate many significant indirect effects that reduce Komodo dragon distribution and abundance. For example, climate and landscape variables are likely to strongly influence the distribution and composition of vegetation communities present within the current Komodo dragon distribution, although potentially with some lag period. The preferred habitat of Komodo dragons currently comprises open deciduous forest, which sustains the complex diversity of prey (i.e., insects through to ungulates) required to support high population abundances (Ariefiandy et al., 2016; Purwandana et al., 2016). A reduction in the quality of vegetation is being observed across much of the current distribution of Komodo dragons as a possible result of recent climate change, causing lower prey availability, contributing to reduced population densities (Ariefiandy, Purwandana, Natali, et al., 2015). Projected increases in temperature under the *Ref Mid* and *Pol Mid* climate scenarios are likely to cause average annual temperatures >27°C at low elevations, resulting in most lowland habitats becoming unsuitable for Komodo dragons within three decades. These temperature increases would likely favor the expansion of more xeric‐adapted open savannah (i.e., low‐quality Komodo dragon habitat) into lowland coastal areas that today comprise open deciduous forest. Such a vegetation transition would result in loss of the Komodo dragon's preferred habitat and associated resources, and would likely have broad‐scale negative population impacts on both Komodo dragons and their prey (Auffenberg, 1981; Heaney, 1991).

Temperature‐related habitat losses are expected to be partially offset by cooler upland habitats becoming more suitable (i.e., warmer; Appendix S3, Figures S3.3 and S3.4). However, the steeper slopes and increased rugosities in the inland/upland areas of the islands are likely to present physical barriers to Komodo dragon range shifts, even if the temperatures and vegetation communities in these areas become more hospitable under climate change. This is based on observations for congeneric species (e.g., Smissen, Melville, Sumner, & Jessop, 2013). Our model indicates that the loss of lowland habitat outweighs any positive effects of the establishment of suitable upland habitat, leading by 2050 to a reduction in the area and quality of suitable habitat, and the number of populations in the metapopulation and their population abundances. The negative impact of the loss of lowland habitats is greatest on the smaller islands (Kode and Motang), as there are fewer moderate‐elevation areas available for refuge.

Our modeling approach is demographically based, not physiologically based. Therefore, it does not directly model the physiological response of Komodo dragons to temperature and distance from coast. However, Komodo dragons are tropical ectotherms that are physiologically specialized with respect to temperature, reducing their capacity for acclimation to climate change (Stillman, 2003). These physiological characteristics mean that the extreme near‐surface temperatures expected in lower, coastal habitats in the future are indeed likely to affect the demography of the species. Thermal tolerance thresholds are likely to be exceeded in open (less vegetated) coastal areas in the future, causing increased costs of thermoregulation and directly influencing individual fitness through decreased metabolic efficiency (Bickford et al., 2010). Therefore, the lower (hotter) coastal areas that the Komodo dragons favor today will become poorer habitat for them in the near future based on our projections. The increased temperature projected by the AOGCM through to 2050 may also directly affect Komodo dragon egg incubation and increase developmental rates. This could cause negative consequences for hatchling emergence, including potential phenological mismatches that may lead to decreased hatchling survival (as shown for other reptiles, e.g., Bickford et al., 2010; Cavallo et al., 2015; Huey & Kingsolver, 1989; Le Galliard, Massot, Baron, & Clobert, 2012; Sinervo et al., 2010).

Our projections of reduction in Komodo dragon occupied range area and metapopulation abundance by 2050 highlight climate‐driven conservation management issues facing Komodo dragons. We suspect that Komodo dragons may be amenable to some of the broad climate change adaptation strategies for biodiversity outlined by Heller and Zavaleta (2009). These include expanding upland reserve networks and translocating dispersal‐limited species. Our work identified populations on the two largest protected islands in KNP (Komodo and Rinca) to fare best under climate change. Therefore, it is imperative that habitats on these islands be managed to reflect their status as important refuges for Komodo dragons (Kearney, Adams, Fuller, Possingham, & Watson, 2018). We suggest that park managers should act now to limit any processes that could exacerbate future habitat loss or degradation in these areas, such as the expansion of ecotourism (Boakes, Fuller, & McGowan, 2018; Hole et al., 2009). Since Komodo dragons, like many island species, are dispersal‐limited (Jessop et al., 2018), translocations may be needed in some cases to facilitate colonization of new favorable environments or climate refugia (Armstrong & Seddon, 2008; Fordham, Akcakaya, et al., 2013; Massot, Clobert, & Ferriere, 2008), particularly if these are located on other islands.

If translocations are required to mitigate extinction risk, they could initially involve the movement of individuals from the most vulnerable populations and habitat patches (e.g., from unprotected, lowland Flores populations and the two smaller protected islands within KNP) into refuge areas with future climate conditions that are more favorable. Further research should focus on using our NPM for Komodo dragons to explore the efficacy of different combinations of conservation intervention strategies (Fordham, Watts, et al., 2012). These future simulations should aim to minimize the extinction threat to Komodo dragons for the smallest economic cost (Wintle et al., 2011).

While our ENM (on which all further demographic modeling was based) had a relatively simple structure with only three environmental predictor variables, it did well at capturing the current‐day ecological niche of the species according to evaluation tests using independent validation data and cross‐validation techniques (Araújo et al., 2019). However, the generality of the ENM is less clear (Araújo et al., 2019). The ENM predicts suitable habitat on Flores for the known distribution of Komodo Dragons on this island, and since data from Flores Island were not used to calibrate the model, these results provide some support that the relationship between model predictors and Komodo dragon presence is transferable and generalizable in space. The inclusion of additional climate parameters and/or direct estimates of prey availability in our ENM (should these data have been available) might have resulted in improved predictions of present‐day habitat suitability (Tikhonov et al., 2020). However, it would not have guaranteed better projections in time or space (Bouchet et al., 2019; Yates et al., 2018), partly because projections from ENMs are highly sensitive to any changes in the covariance structure of predictor variables (Maguire, Nieto‐Lugilde, Fitzpatrick, Williams, & Blois, 2015).

Several other key processes were not considered in our study, which, if accounted for, would most likely synergize with climate change to worsen the future extinction risk of Komodo dragons (Fordham & Brook, 2010). The approach we have used here assumes that biotic interactions remain constant through time in a shifting climate, which is unlikely to be true (Fordham, Akcakaya, et al., 2013). Accounting for biotic interactions directly in the model, and the potential for these interactions to decouple, would likely amplify extinction risk (Lurgi, López, & Montoya, 2012). For example, projected decreases in annual rainfall for eastern Indonesia in the 21st century are likely to increase aridity in the region, leading to a reduction in primary productivity and major structural changes to vegetation communities (Naylor, Battisti, Vimont, Falcon, & Burke, 2007). Such changes would have complex effects on Komodo dragon distribution and abundance, ranging from direct (environmental thermal heterogeneity) to indirect (vegetation composition change and altered fire regimes) effects. Unfortunately, the precipitation variables commonly used in ENMs (e.g., those downloadable from WorldClim2; Fick & Hijmans, 2017) are highly uncertain for our study region and therefore could not be used (see Methods and Appendix S1).

Komodo dragons are sensitive to land‐use change that has occurred in nonprotected habitats on Flores over recent decades (Ciofi & De Boer, 2004). Therefore, ongoing habitat loss and conversion could exacerbate forecast reductions in Komodo dragon abundance and distribution on Flores, which is the only island in the distribution of Komodo dragons where habitat loss continues. Moreover, spatial data were not available for the distribution and abundance of the multiple prey species Komodo dragons feed on across their range, preventing us from directly modeling species interactions and assuming that prey availability is captured implicitly in ENM estimates of habitat suitability (Dormann et al., 2012) and CMR estimates of survival. While we were not able to model the impact of changes in prey availability or current and future land use due to a lack of suitably resolved spatial datasets, this should be prioritized in future research if the spatial layers and data become available.

## 5 CONCLUSIONS AND IMPLICATIONS

Indonesia's biodiversity, with its high levels of endemism and numerous insular populations, is expected to incur severe impacts from global change (Fordham & Brook, 2010; Sodhi et al., 2004). Our projections for an iconic, and highly range‐restricted large‐bodied, predatory lizard support this general assertion and predict that Komodo dragons will be subject to a dramatic population decline and possible extinction due to global warming by 2050 (unless the most optimistic future climate scenarios are realized). The prospect of countering the effects of climate change on Komodo dragons, alongside other agents of rapid global change, is clearly a daunting task facing Indonesia. We strongly advocate that national and provincial conservation agencies act now to address impending climate change impacts on the islands within the Komodo dragon's range, bearing in mind that protected areas on the islands of Komodo and Rinca are likely to provide the best “future‐proofed” habitats for Komodo dragons. Without quick action to mitigate climate change impacts in KNP and on the island of Flores, we risk committing Komodo dragons—a globally iconic species—to extinction.

## CONFLICT OF INTEREST

None declared.

## AUTHOR CONTRIBUTIONS

D.A.F., T.S.J., B.W.B., and A.R.J. conceived the study. A.R.J. developed and validated the models and generated the figures. D.A.F. and B.W.B. guided the development of the models and scenarios. T.M.L.W. provided future climate data and guidance on their use. T.S.J., A.A, D.P., and C.C. provided field data and guidance during interpretation of model outputs and the conservation management context, with input from Y.J.B. and T.S. A.R.J. and T.S.J. wrote the manuscript. All authors commented on drafts of the manuscript.

## Supporting information

Appendix S1‐S4

## Data Availability

Annual projections of habitat suitability for Komodo dragons are on figshare: http://doi.org/10.25909/12768530. Parameters needed to build the coupled niche‐population models are provided in Appendix S1
