# Supplementary material for: Identifying island safe havens to prevent the extinction of the World’s largest lizard from global warming
Source: Ecol Evol. 2020 Sep 15;10(19):10492–507. doi: 10.1002/ece3.6705 (PMC7548163; doi:10.1002/ece3.6705)
Supplement: Supplementary file 1 — Appendix S1‐S4 [file ECE3-10-10492-s001.docx]

**Appendices for: “Identifying island safe havens to prevent the extinction of the World’s largest lizard from global warming”**

**Appendix 1**: Supporting Methods

**Data and ecological niche modelling framework**

Location data

Geographically referenced occurrence data (N = 4028) for Komodo dragons came from historical and recent survey records (detailed in Table S1.1). Presence records were rasterised to a binary grid with a 1-km cell size, resulting in 179 presence cells. Since there is no ‘true-absence’ (detectability calibrated) survey information for Komodo dragons, we randomly generated 1800 pseudo-absences (approximately 10 times the number of presences; Harris et al. 2014) within an area defined by a radial distance of 10 km from all presence cell centroids, based on estimates of Komodo dragon home range (Sastrawan et al. 2002).

**Table S1.1**: **Sources of occurrence data for Komodo dragons** across localities with extant populations in Eastern Indonesia.

| **Authors** | **Records** | **Data collection method** | **Localities** |
| --- | --- | --- | --- |
| Auffenberg (1978) Auffenberg, (1980)  Auffenberg (1981) | 34 | Sightings | Komodo National Park & Flores |
| Ariefiandy *et al* (2013)  Ariefiandy *et al.* (2015)  Jessop et al. (2006)  Jessop *et al.* (2007)  Jessop *et al.* (2008)  Purwandana *et al.* (2014)  Purwandana *et al.* (2015)  Unpublished reports from the Komodo Survival Program | 2295 | Sightings, trapping, camera traps | Komodo National Park & Flores |
| Ciofi & De Boer (2004)  Ciofi unpublished records | 1699 | Trapping, radio tracking | Komodo National Park & Flores |

Climate and environmental predictors

We used the moist adiabatic lapse rate for modelling temperature across the range of the Komodo dragon because 1) temperature is strongly correlated with elevation on tropical mountains, changing rapidly over small horizontal distances (Smith et al. 1987) and 2) robustly validated, long-term, high-spatial-resolution climate data are unavailable for the study area (refer to Figure 4 in Fick et al. 2017). We collated quality-controlled, long-term (1980 to 2014) observational records for annual temperature at sea-level from three weather stations < 300km from the study area and at < 12m elevation (van Oldenborgh 2017). The lengths of the annual temperature time series differed between weather stations. Using these data, we calculated a long-term sea-level temperature baseline of 26.38 °C (n = 56 years of observational data), which is close to that calculated by Harris et al. (2012) for a nearby area of Indonesia using a similar approach and time period. This approach for modelling temperature as a function of elevation is well-established (e.g. see Bush et al. 2004; Raxworthy et al. 2008). To generate a high-resolution raster of average temperature (focused on the beginning of the 21^st^ Century) we combined the sea-level average temperature with high-resolution (250 × 250 m) elevation data derived from the Shuttle Radar Topography Mission (SRTM) (Jarvis et al. 2008).

Justification for excluded model covariates

1. Precipitation

We could not use precipitation as a predictor covariate in the ENMs because there were no good-quality, spatially-resolved data available for the study area. We decided against using WorldClim2 precipitation data because uncertainty in interpolated estimates of precipitation in our study region are very high (see figure 4 in Fick et al. 2017). Unfortunately, weather stations in our study region did not have long-term time series of rainfall data, meaning that spatial estimates could not be generated in-house. As reported in Vimont et al (2010), there are no stations in our study area with >30 years of precipitation data, in fact most weather stations in the region have less than a decade of continuous observations.

The climate data used in Worldclim2 (Fick et al. 2017) are generated using ANUSPLIN, which applies a splining algorithm to weather station data using elevation as a covariate. In the absence of large numbers of well-positioned weather stations (with long time series of observation data), elevation tends to have a strong influence on the results. In these situations, temperature and rainfall estimates are tightly correlated, by virtue of the driving role of elevation in the statistical model. We tested this relationship and present the results in Table S1.2.

Bioclimatic data products at a resolution of 30 arc seconds were downloaded from WorldClim (version 2):

- BIO1 = Annual Mean Temperature
- BIO5 = Max Temperature of Warmest Month
- BIO6 = Min Temperature of Coldest Month
- BIO12 = Annual Precipitation

The bioclimatic variable rasters were cropped to three areas (below) and checked for correlation:

- The full study area (all islands, including Flores)
- Islands in KNP only
- Flores only

The minimum correlation coefficient was -0.73 and the maximum was -0.94, meaning that areas with lower temperature had higher rainfall and vice versa. Areal correlations were highest in KNP – the smallest area of interest. These results support our decision not to include precipitation as a predictor in the ENM due to its extremely strong correlation with temperature (and by proxy, elevation).

**Table S1.2: Correlation coefficients for rainfall and temperature data** from WorldClim for our study region.

| **Extent** | **Temp variable  (long term mean)** | **Rainfall variable  (long term total)** | **Correlation coefficient** |
| --- | --- | --- | --- |
| *Full survey area* | Mean annual temp | Mean annual rainfall | -0.75 |
|  | Mean max temp in warmest month (July) | Mean annual rainfall | -0.74 |
|  | Mean max temp in coolest month (Nov) | Mean annual rainfall | -0.74 |
| *KNP only* | Mean annual temp | Mean annual rainfall | -0.94 |
|  | Mean max temp in warmest month (July) | Mean annual rainfall | -0.89 |
|  | Mean min temp in coolest month (Nov) | Mean annual rainfall | -0.92 |
| *Flores only* | Mean annual temp | Mean annual rainfall | -0.74 |
|  | Mean max temp in warmest month (July) | Mean annual rainfall | -0.73 |
|  | Mean min temp in coolest month (Nov) | Mean annual rainfall | -0.73 |

2. Land-use/habitat class

Spatially explicit land use information for the study region were not appropriate for the development of the Komodo dragon ENM. The data sets we investigated, but rejected are listed below.

The CRISP (250 x 250 m grid cell resolution) data set (Miettinen et al. 2011): In consultation with Komodo dragon experts, we reclassified the 13 original land-use types in the CRISP dataset to a binary classification of suitable or unsuitable habitat for Komodo dragons. This conversion resulted large areas of known unsuitable habitat being classified as suitable habitat. This result is due to the ‘lowland mosaic’ and ‘lowland open’ land use classes in the CRISP dataset incorporating a combination of land uses that are both suitable (e.g., ‘remnants of original vegetation’ and ‘open areas’) and unsuitable (e.g., ‘clearances’ and ‘areas covered by seasonal crops’) for Komodo dragons, leading to poor discrimination of habitat suitability. This is mapped in Figure S1.1 below.


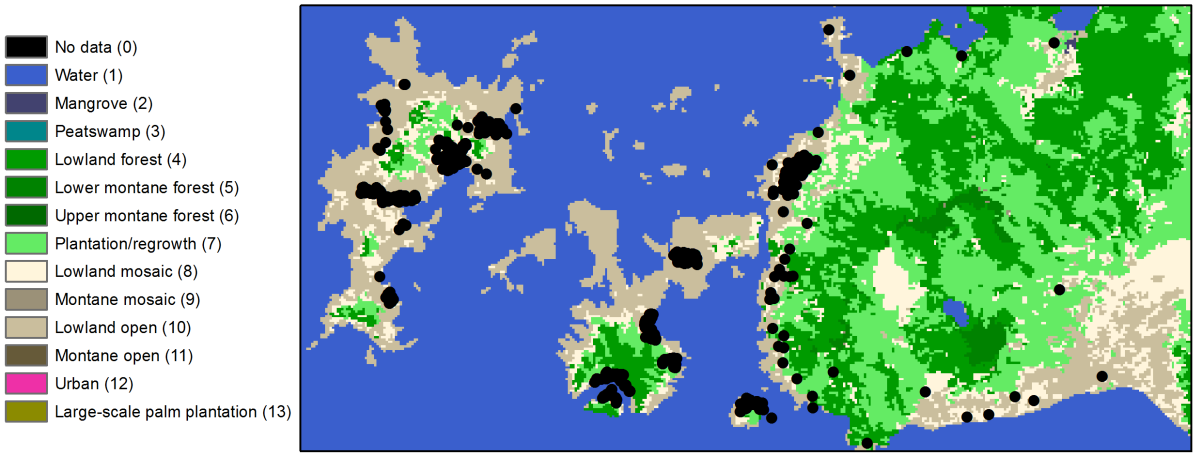


**Figure S1.1: Land use layer from Miettinen et al 2011 with black points indicating records of dragon presence**. Note the high level of overlap between sampled presence locations and the land use classes ‘lowland mosaic’ and ‘lowland open’ (which were deemed by experts to contain areas of both suitable and unsuitable land classes).

Forest cover loss (2000-2012) for Indonesia (Margono et al. 2014): These data layers were not considered appropriate because they only indicate levels of change in forest cover.

Global consensus land-cover (1 x 1 km grid cell resolution) map for biodiversity and ecosystem modelling, with 12 generalised land-use classes (Tuanmu et al. 2014): There were only 2 classes of land cover contained within our study area that had probabilities of presence greater than 50 %: ‘evergreen broadleaf trees’ and ‘cultivated and managed vegetation’ (Figure S1.2). Komodo dragon presences were largely exclusive of the evergreen broadleaf areas and overlapped considerably with the ‘cultivated and managed land use’, despite this being deemed an unsuitable habitat type by the species experts. The ‘cultivated and managed’ land-use category includes a fine-scale mosaic of natural vegetation, crop land and cultivated vegetation, representing both suitable and non-suitable land-use in the same class, which makes this layer too coarse and therefore unsuitable for use in a predictive ENM, or as a habitat suitability mask.


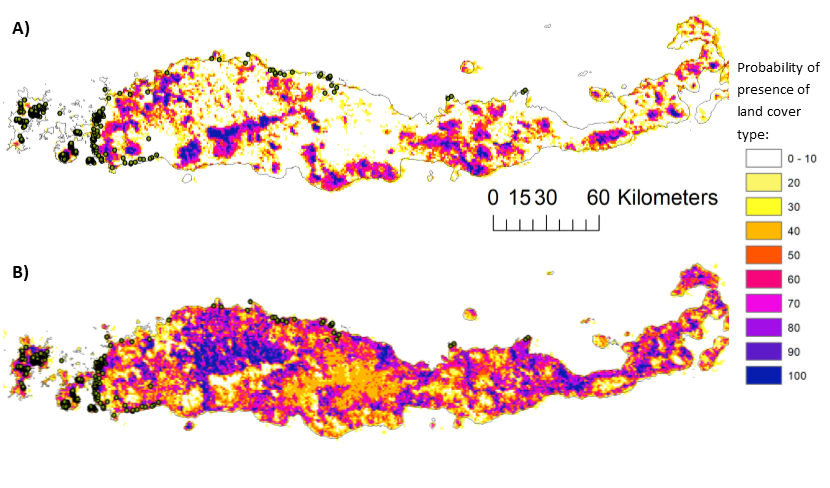


**Figure S1.2:** **Land use layer from Tuanmu et al (2014) with points indicating records of dragon presence**. Note the high probability of presence of the ‘managed and cultivated land cover class at presence locations (which was a category deemed by experts to contain areas of both suitable and unsuitable land classes).

In addition to the above justifications for not including a data layer on land-use, there are also no data available for future predicted land-use/land-use change in the study region. These would be required for carrying out future forecasts from the ENM, unless we assumed that current spatial patterns in land-use would remain static over time (which is unrealistic; Hof et al. 2018). All these issues resulted in us deciding not to include the land-use layer as a covariate in the species distribution models, or as a mask on the model outputs.

**Ecological niche models (ENM)**

We used R (R Core Team 2016) and the package biomod2 (Thuiller et al. 2009) to generate an ENM ensemble of 400 models of Komodo dragon species distributions (100 each of four different modelling algorithms: Generalised Linear Models, Generalised Additive Models, Generalised Boosted-Regression Models and MAXENT). We used a random sample of 80 % of occurrence data to train each model, and the remaining 20 % of the data to evaluate it. Validation of each model’s predictions of Komodo dragon distribution was done using the out-of-sample area under the curve (AUC) of the receiver operating characteristic (ROC) and the true skill statistic (TSS) (Swets 1988; Allouche et al. 2006). We calculated the binary threshold for presence/absence outputs by optimising TSS. Ensemble-averaged outputs of probability of presence (a proxy measure for habitat suitability that can be linked to carrying capacity) were calculated using a consensus estimate from all 400 ENMs with each model’s contribution weighted by its ROC score. We used a separate camera-trap dataset collected in 2013 and 2014 across sites on all five islands (244 presence locations = 67 presences at 1 km × 1 km resolution) to independently evaluate the ensemble-averaged projections of Komodo dragon probability of presence using the Boyce Index (Boyce et al. 2002).

**Capture-mark-recapture models**

We used age-structured Cormack-Jolly-Seber capture-mark-recapture models to estimate age-class and island-specific survival rates for Komodo dragons. Modelling was done using the R package RMark (Laake 2013).

To interpolate missing information on the age of some captured individuals, we used a generalised additive model (GAM) of length-at-age (Laver et al. 2012) to predict age (in years) at initial capture for each individual of unknown age in the CMR dataset based on their sex and snout-vent length. We evaluated the fit of the GAM of length-at-age to the training data (1404 capture events of individuals with known age) using k-fold cross validation, which achieved a mean prediction error of 0.85 years (SD = 0.89 years). Based on the modelled ages, we were able to class the unknown age animals into three age-based life stages (hatchling, juvenile, adult) for the CMR analysis. Komodo dragons on different islands mature at different sizes (Laver et al. 2012), therefore we adjusted the age thresholds for each life stage accordingly (see Table S1.3 and Methods section 2.2 in the main paper).

We calculated year-to-year variance in survival due to demographic stochasticity and subtracted it from the total observed variance in survival to explicitly estimate independent process variance (Akçakaya 2002).

**Table S1.3:** **Age thresholds for life stage classes for each island ‘type’ within Komodo National Park (KNP).** Islands are split into nominally ‘large’ (Komodo and Rinca) and ‘small’ (Nusa Kode and Gili Motang) groups. Boundary ages between life stages (hatchling, juvenile, adult) were based on data from animals in captivity (Walsh et al. 2002), but adjusted upwards (slightly) on advice from experts that Komodo dragons mature later in the wild than in captivity (Mendyk 2015). In addition, Komodo dragons from the small islands have been shown to have different ontogeny to those from the large islands, in that they grow slower and therefore reach maturity at a smaller size on the small islands (Imansyah et al. 2008; Laver et al. 2012). We took this into account in our capture-mark-recapture (CMR) models of survival by adjusting the age thresholds for the small island populations (as per the table below). Note that we only modelled females in our NPM.

| **Small Islands (Kode and Motang & Flores)** | | |
| --- | --- | --- |
|  | ***Male*** | ***Female*** |
| Hatchling | 0-1 yrs | 0-1 yrs |
| Juvenile | 1 - 9 yrs | 1 - 7 yrs |
| Adult | 9 - N yrs | 7 - N yrs |
| **Big islands (Komodo and Rinca)** | | |
|  | ***Male*** | ***Female*** |
| Hatchling | 0-1 yrs | 0-1 yrs |
| Juvenile | 1 - 15 yrs | 1 - 9 yrs |
| Adult | 15 - N yrs | 9 - N yrs |

**Niche-population model (NPM) framework and parameterisation**

Survival rates

Hatchling survival (from 0 – 12 months) in the stage-structured matrix model was constant across islands. Its value was based on estimates for Rinca, where the most extensive data on hatchlings exists. Survival for juveniles and adults in the matrix model varied with location based on CMR model estimates for each age class (see Results in main text). Since there were no CMR data from Flores, and because the abundance of Komodo dragons is declining on this island (Ciofi & De Boer, 2004; Ariefiandy et al., 2015), we used the average of the lowest apparent survival estimates for the small islands of Motang and Kode to model survival in Flores sub-populations outside of reserves. The within-reserve sub-populations on Flores were modelled using the highest estimate of survival rate (from Rinca), to account for the assumed benefits of being within a managed protected area.

Fecundity

Fecundity was estimated by multiplying the proportion of reproductively active females, mean clutch size, hatch success rate and sex ratio at birth: Where reproductively active females = 80%; mean clutch size = 18 eggs; hatching success rate = 60%; and sex ratio = 1:1 (Thompson et al., 2001; Walsh et al., 2002; Purwandana, 2007). Although Komodo dragons tend to have a relatively stable clutch size, the proportion of females breeding each year varies (Walsh et al., 2002; Purwandana, 2007), so we used a coefficient of variation of 0.15 for the modelled fecundity parameter, based on expert advice.

Density dependence

The primary factor limiting Komodo dragon population size is prey availability (Purwandana et al., 2015). Thus, density dependence was modelled using a Ricker-logistic equation, which assumes worsening returns as the population of juveniles and adults increases and the amount of resources per individual decreases (Akcakaya et al. 2005). Hatchlings were excluded from the density-dependence calculation because they have an arboreal life history and are not in direct competition for the same resources as the older life stages. Rmax, the maximum rate of intrinsic population growth (per capita birth rate minus the per capita death rate) when the population has unlimited resources, was estimated at 1.18 based on a Pradel CMR estimate for Komodo dragon populations on Komodo and Rinca islands (Purwandana et al., 2015).

Dispersal

We constrained dispersal to juveniles and adults (Imansyah, 2006) and modelled the proportion of dispersing individuals among populations using a negative exponential function. Based on CMR, radio telemetry and genetic relatedness studies, we estimate (in our model) that approximately 99 % of animals move less than 1 km (i.e., stayed within a single grid cell), ~1 % moved more than 1 km, of which ~0.05 % moved up to 8 km (Ciofi & Bruford, 1999) (T. Jessop unpublished dispersal data; Fig. S1.3).

**Figure S1.3**: **The dispersal by distance function used in the Komodo dragon metapopulation model** to represent adult and juvenile dispersal. The function was determined using unpublished annual displacement data from (Jessop et al. 2018), supported by information on genetic relatedness between populations within and between islands (Ciofi et al. 1999a; Ciofi et al. 1999b). The data from T. Jessop indicate that around 35 % of the individuals in the CMR study did not move away from their capture site during the 10-year study period. The maximum displacement distance recorded was ~8km.

Carrying capacity

We multiplied the ENM projections of habitat suitability with a time-invariant layer of ‘management benefit’, where cells on small islands and outside reserves on Flores = 1, whereas cells on large islands and those inside reserves on Flores = 1.9. This was done because habitat quality is approximately 90 % better on the two larger islands within KNP (Rinca and Komodo) due to factors such as prey availability (Purwandana et al. 2015), which we could not explicitly model in our ENM due to a lack of spatial data on prey distribution and quality. A similar increase in habitat quality is expected inside Flores reserves, although no quantitative data are available to test this assumption. The carrying capacity (*K*) of each habitat patch was then determined by the ENM-predicted habitat suitability values at each time step using:

**Equation 1**: *K* = *ths* * 5 * *gte* (*noc*, 2)

where ‘*ths*’ is the sum of the ENM-predicted habitat suitability of all cells within the patch, with a minimum number of cells (‘*noc*’) of 2 required per patch. For cases where *noc* < 2, *K* was assumed to be zero based on a ‘greater than’ function (*gte*). We used a multiplier to convert habitat suitability (a proportional value based on the ENM estimates of probability of presence in a grid cell, multiplied by a ‘management benefit’ where applicable – see above) into carrying capacity: *K*, the number of individuals supported by the habitat in a grid cell. The multiplier was set at 5, based on this achieving the best approximation of recent island-based and total-KNP upper abundance estimates (Jessop et al. 2007; Purwandana et al. 2014; Purwandana et al. 2015). Table S1.4 shows these published upper abundance estimates (halved to represent only the females, as we used a female only model) and the initial abundance estimates for each island within KNP (and the KNP total) from the RAMAS baseline models. The RAMAS model parameterisation tended to slightly overestimate the carrying capacity on each island, with a greater discrepancy on the smaller two islands (Kode and Motang).

**Table S1.4:** A comparison of published island-level *K* estimates for Komodo dragons inside KNP and the estimated carrying capacity from the baseline RAMAS models.

|  | **RAMAS baseline *K*** | **Published estimates** |
| --- | --- | --- |
| **KNP total** | 1297 | 1230 |
| **Komodo** | 727 | 583 |
| **Rinca** | 462 | 592 |
| **Motang** | 59 | 22 |
| **Kode** | 49 | 26 |

Abundance in the NPM’s first annual time step was modelled as being equal to 80% of patch carrying capacity (Fordham et al. 2012). We set a minimum viable sub-population (patch) size of 4 females, based on estimates of minimum density (Purwandana et al. 2014), below which extirpation occurs. Population patches outside of the two protected areas on Flores (Wae Wuul and Wolo Tado) were given initial abundance values of 0 and small patches of habitat on Flores (≤ 2 cells) were deemed unable to support sub-populations (parameterised with carrying capacity, *K*, of 0). These settings meant that Komodo dragons could disperse through un-protected, fragmented habitat patches on Flores; but that these areas could generally not support resident sub-populations. This reflects the sparse and fragmented dragon populations on Flores, and the benefits of being within a protected area (Ariefiandy et al. 2015). Uncertainty in carrying capacity, associated with the association between ENM projections of Komodo dragon habitat suitability and upper abundance, was accounted for in the NPM model simulations, by varying the value of *K* for each population patch by ± 20 % (see Table S1.5). We were unable to directly assess the effect of spatiotemporal uncertainty in ENM projections due to an absence of independent observation data

Stochasticity

Variability in environmental conditions (environmental stochasticity) may lead to unpredictable impacts on Komodo dragon survival and reproduction. We assumed the effect of environmental stochasticity on Komodo dragon vital rates was spatially correlated between habitat patches (sub-populations) in our modelled landscape. We used a negative exponential function to model variability in environmental conditions based on rainfall variability in the region (Gregory et al. 2014).

**NPM sensitivity analysis**

We undertook a sensitivity analysis to assess the most influential population model parameters using SARDM (Fordham et al. 2016). We ran this sensitivity analysis on two of the climate change scenarios (RCP 8.5 and RCP 2.6), both with mid-range climate sensitivity and aerosol forcing (‘Policy-Mid’ and ‘Reference-Mid’). We varied fecundity, Rmax, survival, carrying capacity and dispersal across set parameter ranges (Table S1.5) using 200 Latin hypercube samples. We used the built-in analysis tool to explore the relative importance of different parameter values on EMA, final population size (for simulation runs where the population persists) and final number of occupied patches using general linear models (GLM) (Fordham et al. 2016). We show normalised, standardized regression coefficients (SRC = coef/S.E.) for each term in the full GLM (i.e. the model including all variables), re-scaled by dividing by the sum of all SRCs. The SRC is particularly useful as it provides a relative (dimensionless)—and therefore directly cross-comparable—metric of parameter sensitivity(McCarthy et al. 1995).

**Table S1.5: Parameter estimates used in the coupled Niche-Population Models** (NPM; female only), including the ranges used to model stochasticity in RAMAS GIS and for sensitivity analysis in SARDM (Fordham et al. 2016). Justification of each estimate is provided along with sources of data or supporting information relevant to each parameter.

| **Parameter** | **Value (RAMAS)** | **CV (RAMAS)** | **Range (SARDM)** | **Distribution (SARDM)** | **Description and source of information used to inform estimates** |
| --- | --- | --- | --- | --- | --- |
| *Age at maturity* |  |  |  |  | Island-based ontogeny (Laver et al. 2012) - see female values in Table S1.3 |
| Large islands | 9 | — | — | — |  |
| Small islands & Flores | 7 | — | — | — |  |
| *Survival rates* |  | 0.22 | 83 - 117 % | Beta distribution.  Alpha = 2, Beta = 2 |  |
| Hatchling | 0.2 |  |  |  | CMR models of first year survival;(Purwandana 2007); (IUCN 2017). |
| Juvenile | Vary by island |  |  |  | Stage and island-specific estimates of survival from the CMR models (see results in main paper and Table S1.3). Process variance around survival rates was calculated as per (Akçakaya 2002) and generalised across all populations. |
| Adult | Vary by island |  |  |  |  |
| *Rmax* (for all populations) | 1.18 | — | Lower = 1, Scale = 0.13, Sigma = 0.4 | Lognormal distribution | Pradel CMR estimate for Komodo dragon populations on Komodo and Rinca islands (Purwandana et al. 2015). |
| *Dispersal* (adults and juveniles only) | Varying, based on a dispersal distance function (*a* = 0.1, *b* = 0.35, *c* = 0.30 and *Dmax* = 8.0) and dependent on target population’s carrying capacity (*K*) | — | 90 - 110% | Uniform distribution | Parameterisation of the dispersal function was based on capture-mark-recapture (CMR) analyses, radio telemetry and genetic relatedness studies. The function results in approximately 99 % of animals moving less than 1 km at each time step (i.e., most stay within a single grid cell), ~1 % move more than 1 km, of which ~0.05 % move up to a maximum dispersal distance of 8 km (Ciofi et al. 1999b; Jessop et al. 2018) |
| *Fecundity* (adults only) | 4.32 | 0.15 | Lower bound = 0.8, scale = 0.19, Sigma = 0.3 | Lognormal distribution | Fecundity calculated as mean clutch size * sex ratio at birth * proportion of reproductively active females * hatch success rate (Fordham et al. 2008). Variance around fecundity was based on expert opinion (no empirical data available). |
| Breeding frequency | 0.8 | — | — | — | Purwandana (2007) |
| Clutch size | 18 | — | — | — | Wild and captive populations: (Purwandana 2007); (Birchard et al. 1995); (Thompson et al. 2001); (Walsh et al. 1993); (Pether et al. 2007); (Purwandana 2007), (Sunter 2008) |
| Hatching success | 0.6 | — | — | — | Data from captive populations: (Birchard et al. 1995), (Thompson et al. 2001); (Walsh et al. 1993); (Pether et al. 2007), (Purwandana 2007), (Sunter 2008). |
| Hatchling sex ratio | 1:1 | — | — | — | Captive populations:(Walsh et al. 2002); Wild populations: (Purwandana 2007). |
| *Carrying capacity (K)* | Patch-based values derived from ENM predictions of habitat suitability | — | 80 – 120% | Triangular | The carrying capacity of each habitat patch was determined by the ENM-predicted habitat suitability values at each time step (see Appendix 1 for equation). A multiplier of *K* was applied to populations within large protected areas (Purwandana et al. 2015) |
| *Abundance* | Patch-based values, related to carrying capacity (*K*) | 0.2 | — | — | The abundance in each habitat patch was limited by the patch’s carrying capacity (*K*). Initial abundance in each patch at the start of each model simulation was set at  80 % of *K*. |
| *Density dependence* (affects adult and juvenile survival) | Ricker logistic (scramble) model based on each sub-population’s carrying capacity | — | — | — | A scramble competition model for density dependence was used, based on food being the primary factor limiting abundance within habitat patches (Jessop et al. 2007; Laver et al. 2012). Density dependence affected survival rates for juveniles and adults, but not hatchlings which are arboreal and therefore not competing for the same resources. |
| *Demographic and environmental stochasticity* | Lognormal distribution | — | — | — | We used RAMAS GIS in-built stochasticity functionality, based on a lognormal function and using within-population correlation for fecundity, survival and carrying capacity (Akcakaya et al. 2005) |
| *Environmental correlation* | Correlation-distance function: a = 0.35, b = 500, c = 1.0 | — | — | — | Adapted from Gregory et al (2014), based on rainfall data from 50 weather stations within the study region (centred on Borneo, which is north of our islands). |

**References**

Akcakaya, H. R. and W. T. Root (2005). RAMAS GIS: Linking landscape data with population viability analysis (version 5), Applied Biomathematics, Setauket, New York.

Akçakaya, R. H. (2002). "Estimating the variance of survival rates and fecundities." Animal Conservation **5**(04): 333-336.

Allouche, O., A. Tsoar and R. Kadmon (2006). "Assessing the accuracy of species distribution models: prevalence, kappa and the true skill statistic (TSS)." Journal of Applied Ecology **43**(6): 1223-1232.

Ariefiandy, A., D. Purwandana, C. Natali, M. J. Imansyah, M. Surahman, T. S. Jessop and C. Ciofi (2015). "Conservation of Komodo dragons *Varanus komodoensis* in the Wae Wuul nature reserve, Flores, Indonesia: a multidisciplinary approach." International Zoo Yearbook **49**: 67-80.

Birchard, G. F., T. Walsh, R. Rosscoe and C. L. Reiber (1995). "Oxygen Uptake by Komodo Dragon (Varanus komodoensis) Eggs: The Energetics of Prolonged Development in a Reptile." Physiological Zoology **68**(4): 622-633.

Boyce, M. S., P. R. Vernier, S. E. Nielsen and F. K. A. Schmiegelow (2002). "Evaluating resource selection functions." Ecological Modelling **157**(2-3): 281-300.

Bush, M. B., M. R. Silman and D. H. Urrego (2004). "48,000 years of climate and forest change in a biodiversity hot spot." Science **303**(5659): 827-829.

Ciofi, C., M. A. Beaumont, I. R. Swingland and M. W. Bruford (1999a). "Genetic divergence and units for conservation in the Komodo dragon *Varanus komodoensis*." Proceedings of the Royal Society B - Biological Sciences **266**(1435): 2269-2274.

Ciofi, C. and M. W. Bruford (1999b). "Genetic structure and gene flow among Komodo dragon populations inferred by microsatellite loci analysis." Molecular Ecology **8**(12): S17-S30.

Fick, S. E. and R. J. Hijmans (2017). "WorldClim 2: new 1-km spatial resolution climate surfaces for global land areas." International Journal of Climatology **37**(12): 4302-4315.

Fordham, D. A., A. Georges and B. W. Brook (2008). "Indigenous harvest, exotic pig predation and local persistence of a long-lived vertebrate: managing a tropical freshwater turtle for sustainability and conservation." Journal of Applied Ecology **45**(1): 52-62.

Fordham, D. A., S. Haythorne and B. W. Brook (2016). "Sensitivity Analysis of Range Dynamics Models (SARDM): Quantifying the influence of parameter uncertainty on forecasts of extinction risk from global change." Environmental Modelling & Software **83**: 193-197.

Fordham, D. A., H. Resit Akçakaya, M. B. Araújo, J. Elith, D. A. Keith, R. Pearson, T. D. Auld, C. Mellin, J. W. Morgan, T. J. Regan, M. Tozer, M. J. Watts, M. White, B. A. Wintle, C. Yates and B. W. Brook (2012). "Plant extinction risk under climate change: are forecast range shifts alone a good indicator of species vulnerability to global warming?" Global Change Biology **18**(4): 1357-1371.

Gregory, S. D., M. Ancrenaz, B. W. Brook, B. Goossens, R. Alfred, L. N. Ambu and D. A. Fordham (2014). "Forecasts of habitat suitability improve habitat corridor efficacy in rapidly changing environments." Diversity and Distributions **20**(9): 1044-1057.

Harris, B. C. J., D. A. Fordham, P. A. Mooney, L. P. Pedler, M. B. Araújo, D. C. Paton, M. G. Stead, M. J. Watts, H. Reşit Akçakaya and B. W. Brook (2012). "Managing the long-term persistence of a rare cockatoo under climate change." Journal of Applied Ecology **49**(4): 785-794.

Harris, D. B., S. D. Gregory, B. W. Brook, E. G. Ritchie, D. B. Croft, G. Coulson and D. A. Fordham (2014). "The influence of non-climate predictors at local and landscape resolutions depends on the autecology of the species." Austral Ecology **39**(6): 710-721.

Hof, C., A. Voskamp, M. F. Biber, K. Böhning-Gaese, E. K. Engelhardt, A. Niamir, S. G. Willis and T. Hickler (2018). "Bioenergy cropland expansion may offset positive effects of climate change mitigation for global vertebrate diversity." Proceedings of the National Academy of Sciences **115**(52): 13294-13299.

Imansyah, M. J., T. S. Jessop, C. Ciofi and Z. Akbar (2008). "Ontogenetic differences in the spatial ecology of immature Komodo dragons." Journal of Zoology **274**(2): 107-115.

IUCN. (2017). "The IUCN Red List of Threatened Species. Version 2017-2." Retrieved 5th November, 2017, 2017, from <http://www.iucnredlist.org>.

Jarvis, A., H. I. Reuter, A. Nelson and E. Guevara (2008). "Hole-filled SRTM for the globe Version 4." available from the CGIAR-CSI SRTM 90m Database (<http://srtm>. csi. cgiar. org).

Jessop, T. S., A. Ariefiandy, D. Purwandana, C. Ciofi, J. Imansyah, J. Benu, D. A. Fordham, D. Forsyth, R. Mulder and B. L. Phillips (2018). "Exploring mechanisms and origins of reduced dispersal in island Komodo dragons." Proceedings of the Royal Society B: Biological Sciences **18**(29).

Jessop, T. S., T. Madsen, C. Ciofi, M. J. Imansyah, D. Purwandana, H. Rudiharto, A. Arifiandy and J. A. Phillips (2007). "Island differences in population size structure and catch per unit effort and their conservation implications for Komodo dragons." Biological Conservation **135**(2): 247-255.

Laake, J. L. (2013). RMark: An R Interface for Analysis of Capture-Recapture Data with MARK. AFSC Processed Report 2013-01. Seattle, WA, Alaska Fisheries Science Centre, NOAA**:** 25p.

Laver, R. J., D. Purwandana, A. Ariefiandy, J. Imansyah, D. Forsyth, C. Ciofi and T. S. Jessop (2012). "Life-History and Spatial Determinants of Somatic Growth Dynamics in Komodo Dragon Populations." PLoS ONE **7**(9): e45398.

Margono, B. A., P. V. Potapov, S. Turubanova, F. Stolle and M. C. Hansen (2014). "Primary forest cover loss in Indonesia over 2000-2012." Nature Clim. Change **4**(8): 730-735.

McCarthy, M. A., M. A. Burgman and S. Ferson (1995). "Sensitivity analysis for models of population viability." Biological Conservation **73**(2): 93-100.

Mendyk, R. W. (2015). "Life expectancy and longevity of varanid lizards (Reptilia:Squamata:Varanidae) in North American zoos." Zoo Biology **34**(2): 139-152.

Miettinen, J., C. Shi, W. J. Tan and S. C. Liew (2011). "2010 land cover map of insular Southeast Asia in 250-m spatial resolution." Remote Sensing Letters **3**(1): 11-20.

Pether, J. and G. Visser (2007). "The first breeding of Komodo dragons as a result of the European Endangered Species Breeding Programme (E.E.P.)." Mertensiella 16. Advances in Monitor Research III: 430 - 440.

Purwandana, D. (2007). Nesting activity and spatial ecology of female Komodo Dragons (*Varanus komodoensis*) in the Komodo National Park, Indonesia. MSc, Fakulti Sains Dan Teknologi, Universiti Kebangsaan Malaysia.

Purwandana, D., A. Ariefiandy, M. J. Imansyah, C. Ciofi, D. M. Forsyth, A. M. Gormley, H. Rudiharto, A. Seno, D. A. Fordham, G. Gillespie and T. S. Jessop (2015). "Evaluating environmental, demographic and genetic effects on population-level survival in an island endemic." Ecography: 1060 - 1070.

Purwandana, D., A. Ariefiandy, M. J. Imansyah, H. Rudiharto, A. Seno, C. Ciofi, D. A. Fordham and T. S. Jessop (2014). "Demographic status of Komodo dragons populations in Komodo National Park." Biological Conservation **171**(0): 29-35.

R Core Team (2016). R: A language and environment for statistical computing. R Foundation for Statistical Computing. Vienna, Austria.

Raxworthy, C. J., R. G. Pearson, N. Rabibisoa, A. M. Rakotondrazafy, J. B. RAMANAMANJATO, A. P. Raselimanana, S. Wu, R. A. Nussbaum and D. A. Stone (2008). "Extinction vulnerability of tropical montane endemism from warming and upslope displacement: a preliminary appraisal for the highest massif in Madagascar." Global Change Biology **14**(8): 1703-1720.

Sastrawan, P. and C. Ciofi (2002). Population distribution and home range. Komodo Dragons: Biology and Conservation. J. B. Murphy, C. Ciofi, C. de la Panouse and T. Walsh. Washinton, DC, Smithsonian Books**:** 324.

Smith, A. P. and T. P. Young (1987). "Tropical alpine plant ecology." Annual Review of Ecology and Systematics **18**(1): 137-158.

Sunter, G. (2008). "Management and reproduction of the Komodo dragon Varanus komodoensis Ouwens 1912 at ZSL London Zoo." International Zoo Yearbook **42**(1): 172-182.

Swets, J. (1988). "Measuring the accuracy of diagnostic systems." Science **240**(4857): 1285-1293.

Thompson, G. G., E. R. Pianka and J. McEachran (2001). "Allometry of clutch and neonate sizes in monitor lizards (Varanidae: Varanus)." Copeia **2001**(2): 443-458.

Thuiller, W., B. Lafourcade, R. Engler and M. B. Araújo (2009). "BIOMOD – a platform for ensemble forecasting of species distributions." Ecography **32**(3): 369-373.

Tuanmu, M.-N. and W. Jetz (2014). "A global 1-km consensus land-cover product for biodiversity and ecosystem modelling." Global Ecology and Biogeography.

van Oldenborgh, G. J. (2017). "KNMI Climate Explorer." Retrieved 25/09/2017, 2017, from <http://climexp.knmi.nl/allstations.cgi?id=someone@somewhere&climate=sealevel_pressure&n=12>.

Vimont, D. J., D. S. Battisti and R. L. Naylor (2010). "Downscaling Indonesian precipitation using large-scale meteorological fields." International Journal of Climatology **30**(11): 1706-1722.

Walsh, T., D. Chiszar, G. F. Birchard and K. A. Tirtodiningrat (2002). Captive Management and Growth. Komodo Dragons: Biology and Conservation. J. B. Murphy, C. Ciofi, C. de la Panouse and T. Walsh. Washinton, DC, Smithsonian Books**:** 324.

Walsh, T., R. Rosscoe and G. F. Birchard (1993). "Dragon tales, the history, husbandry, and breeding of Komodo monitors at the National Zoological Park." Vivarium **4**(6): 23-26.

**Appendix 2**: Plausible climate future scenarios (n = 6) for the geographic distribution of Komodo dragons


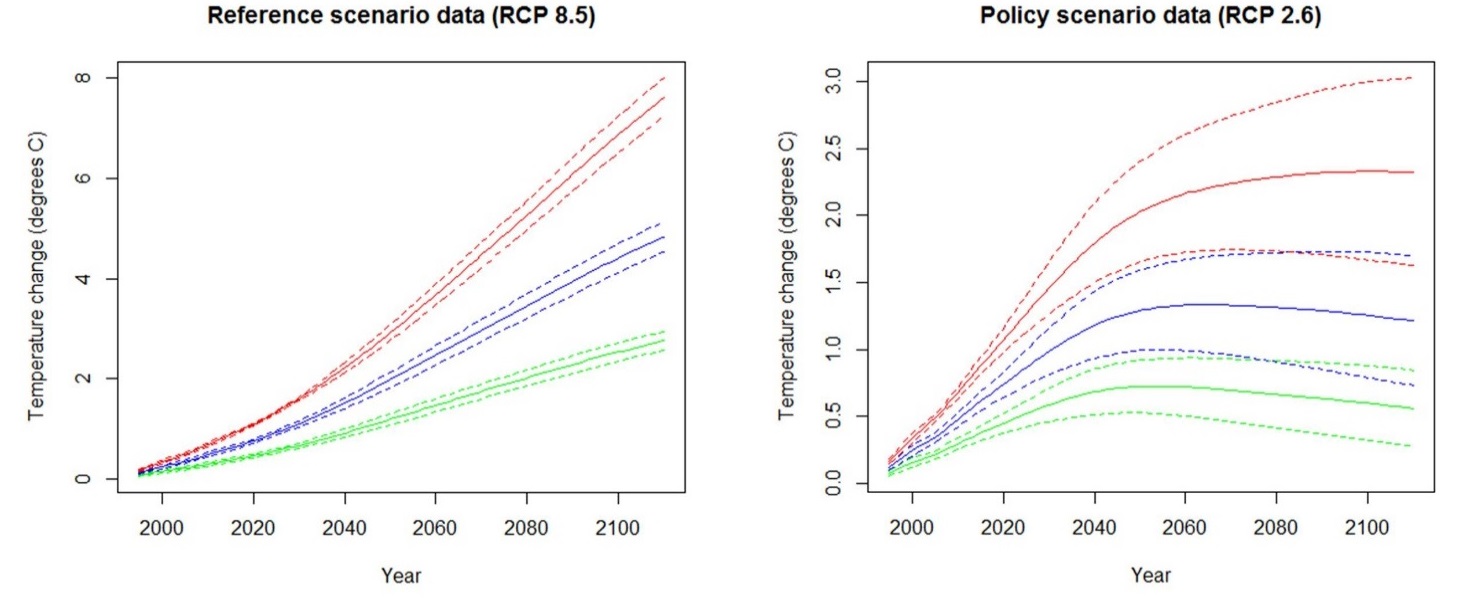


**Figure S2.1**: **Climate model ensemble-averaged projections of temperature increase for the range-wide distribution of the Komodo dragon** under two contrasting global greenhouse gas emissions scenarios. Left: A no new emissions policy (reference, *Ref*) scenario with no stabilisation of greenhouse gas emissions (RCP 8.5). Right: A corresponding stringent global greenhouse gas emissions (policy, *Pol*) scenario that achieves stabilisation of greenhouse gas emissions (RCP 2.6). Both emissions scenarios account for structural climate model uncertainty around climate sensitivity and forcing from aerosols using three parameterisations: green = low climate sensitivity and aerosol forcing; blue = median range climate sensitivity and aerosol forcing; red = high climate sensitivity and aerosol forcing (see Table 1 in main text for parameterisation details). Note different Y axes on the two plots.


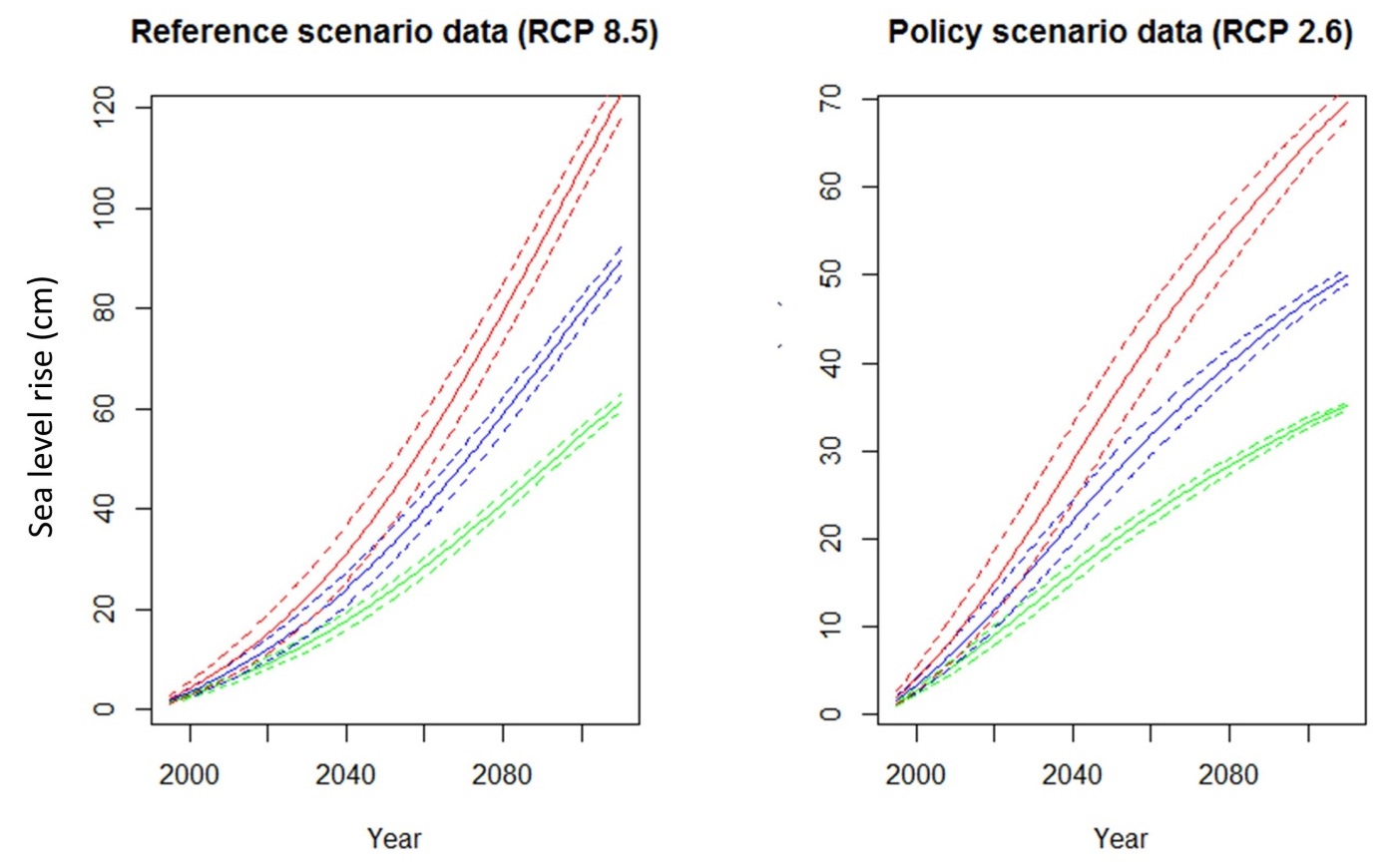


**Figure S2.2**: **Climate model ensemble-averaged projections of sea-level rise for the range-wide distribution of the Komodo dragon** under two contrasting global greenhouse gas emissions scenarios. Left: A no new emissions policy (reference, *Ref*) scenario with no stabilisation of greenhouse gas emissions (RCP 8.5). Right: A corresponding stringent global greenhouse gas emissions (policy, *Pol*) scenario that achieves stabilisation of greenhouse gas emissions (RCP 2.6). Both emissions scenarios account for structural climate model uncertainty around climate sensitivity and forcing from aerosols using three parameterisations: green = low climate sensitivity and aerosol forcing; blue = middle of the range climate sensitivity and aerosol forcing; red = high climate sensitivity and aerosol forcing (see Table 1 in main text for parameterisation details). Note different Y axes on the two plots.

**Appendix 3**: Results from ecological niche models (ENM)


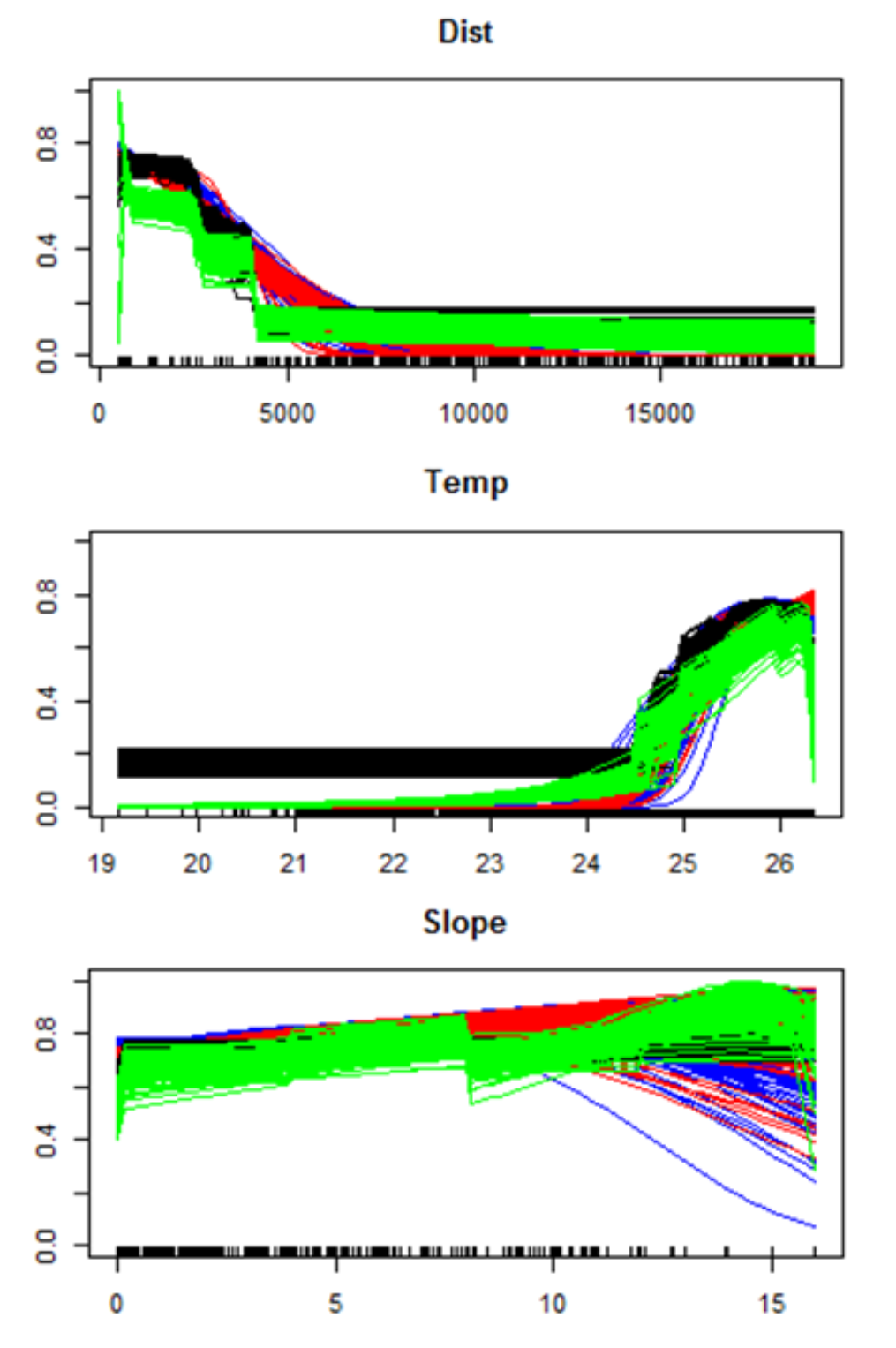


**Figure S3.1**: **Ensemble Ecological Niche Model (ENM) response curves for the three model predictor variables.** Top: Distance from coast (Dist; meters), middle: average annual temperature (Temp; °C) and bottom: topographical slope (Slope; number of constituent cells with slope >75^th^ percentile). Coloured lines indicate the 4 different modelling algorithms used in the ensemble model (n = 100 models per algorithm). Rug plot along the horizontal axis shows the distribution of presence points in variable space.

**Table S3.1:** **Ecological Niche Model (ENM) projections.** Changes in availability of suitable habitat for Komodo dragons in 2050 (from a baseline focused on 2010) under six different future climate scenarios.

| **Future climate scenario** | **Change in area of suitable habitat (%)** | **Change in area of suitable habitat (km^2^)** |
| --- | --- | --- |
| *Pol low* | -8.4 | -223 |
| *Pol mid* | -30.2 | -736 |
| *Pol high* | -71.5 | -1604 |
| *Ref low* | -31.1 | -821 |
| *Ref mid* | -64.4 | -1568 |
| *Ref high* | -87 | -1952 |


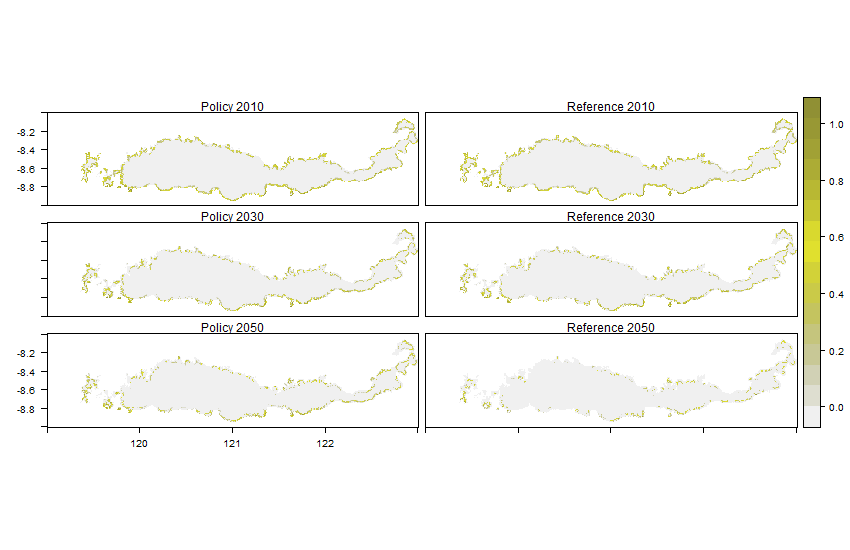


**Figure S3.2**: **Maps of Komodo dragon habitat suitability** used in the coupled niche-population model. The maps show three snapshots in time: beginning (2010), midway (2030) and at the end (2050) of the simulation for the Pol-Mid (left) and Ref-Mid (right) climate change scenarios.

**
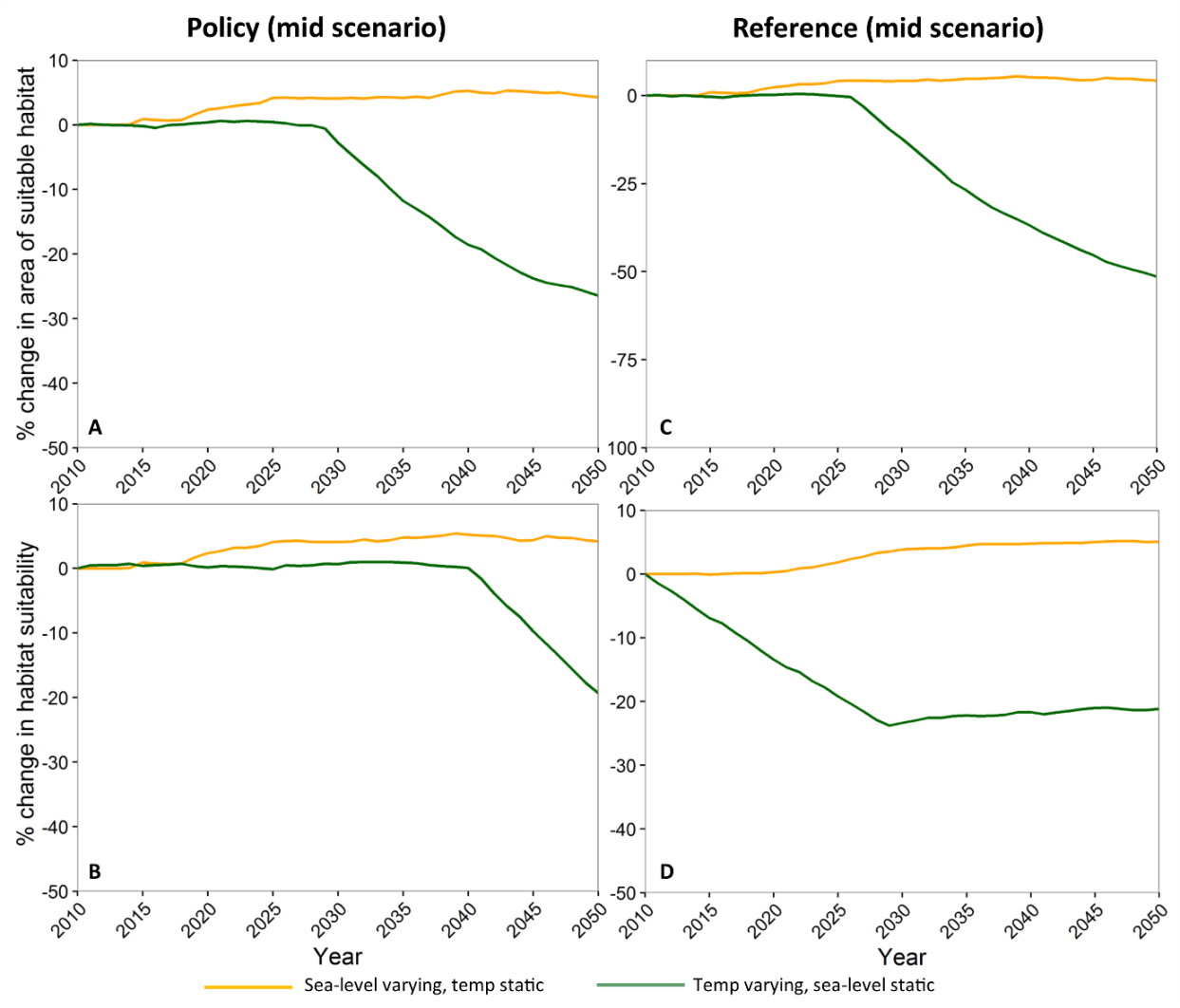

Figure S3.3**: **Ensemble averaged ecological niche model (ENM) projections of change in the area of suitable habitat and average suitability** with varying sea-level and static (present-day) temperature (yellow), and varying temperature and static sea level (green). Projections are shown for two contrasting plausible future climate scenarios: **C & D**) a no new climate-policy reference (*Ref*) scenario (with no greenhouse gas emission stabilisation, [RCP 8.5]); **A & B**) and a corresponding stringent global greenhouse gas emissions policy (*Pol*) scenario (leading to stabilisation, [RCP 2.6]). Both with mid-range climate sensitivity and aerosol forcing. The slight positive effect of sea level rise (< 5 %) is driven by positive effect of a reduced distance to coast (and associated habitat changes) until around 2050, after which sea level rise causes a steady decline in habitat suitability and available area (see Fig. S3.3).


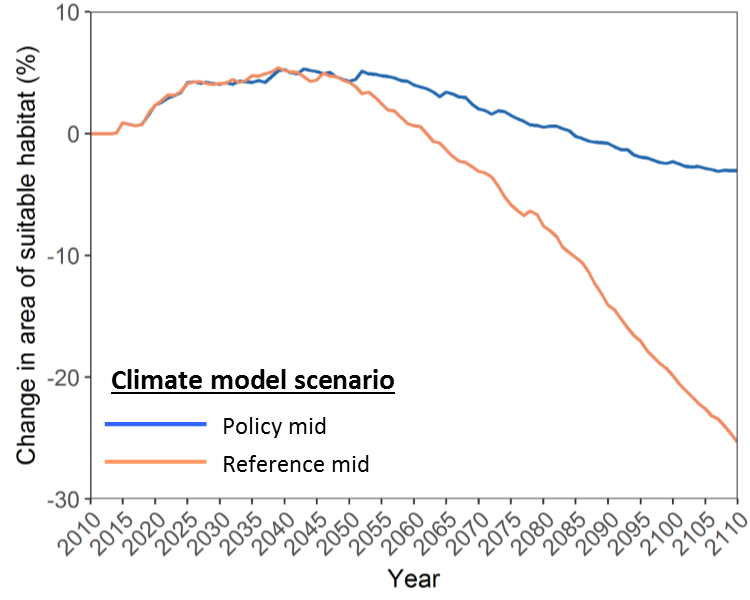


**Figure S3.4**: **Ensemble averaged ENM projections of change in the area of suitable habitat with varying sea-level and static (present-day) temperature to 2110**. Projections are shown for two contrasting future climate scenarios. Blue line = no new climate-policy reference (*Ref*) scenario (with no greenhouse gas emission stabilisation, [RCP 8.5]). Orange line = a corresponding stringent global greenhouse gas emissions policy (*Pol*) scenario (leading to stabilisation, [RCP 2.6]). Both with mid-range climate sensitivity and aerosol forcing.

**Appendix 4:** Results from Niche Population Models (NPM)

**Table S4.1: Outputs from island-based population models (NPMs) for each plausible future climate scenario (n = 6).** Change (Δ) in abundance and patch occupancy are based on the 50th percentile from 200 models with varying parameter values (each run with 1000 stochastic simulations). Lower and upper confidence intervals indicated by the 5th and 95th percentiles respectively. Expected minimum abundance (EMA) is shown as the 50th percentile. See Methods in main text for more details.

| **Island** | **Future climate scenario** | **Δ abundance** | **Δ patch occupancy** | **EMA** |
| --- | --- | --- | --- | --- |
|  | *Ref low* | -100 (-100, -100) | -100 (-100, -100) | 0 |
|  | *Ref mid* | -100 (-100, -100) | -100 (-100, -100) | 0 |
| **Flores** | *Ref high* | -100 (-100, -100) | -100 (-100, -100) | 0 |
|  | *Pol low* | -34 (-53, -15) | -41 (-57, -30) | 35 |
|  | *Pol mid* | -100 (-100, -100) | -100 (-100, -100) | 0 |
|  | *Pol high* | -100 (-100, -100) | -100 (-100, -100) | 0 |
|  | *Ref low* | -92 (-94, -90) | -87 (-88, -85) | 54 |
|  | *Ref mid* | -91 (-92, -85) | -86 (-89, -82) | 63 |
| **Komodo** | *Ref high* | -98 (-98, -97) | -95 (-96, -94) | 18 |
|  | *Pol low* | -31 (-42, -17) | -15 (-22, -8) | 438 |
|  | *Pol mid* | -80 (-83, -75) | -58(-61, -52) | 145 |
|  | *Pol high* | -92 (-94, -91) | -86 (-88, -85) | 55 |
|  | *Ref low* | -99 (-99, -98) | -98 (-99, -97) | 4 |
|  | *Ref mid* | -96 (-98, -94) | -93 (-93, -92) | 26 |
| **Rinca** | *Ref high* | -100 (-100, -100) | -100 (-100, -100) | 0 |
|  | *Pol low* | -23 (-34, -11) | -41 (-45, -36) | 300 |
|  | *Pol mid* | -72 (-76, -68) | -62 (-62, -61) | 140 |
|  | *Pol high* | -99 (-100, -98) | -98 (-99, -97) | 3 |
|  | *Ref low* | -100 (-100, -100) | -100 (-100, -100) | 0 |
|  | *Ref mid* | -100 (-100, -100) | -100 (-100, -100) | 0 |
| **Kode** | *Ref high* | -100 (-100, -100) | -100 (-100, -100) | 0 |
|  | *Pol low* | 3 (-29, 29) | -10 (-30, 0) | 7 |
|  | *Pol mid* | -6(-29, 11) | -10 (-30, 0) | 7 |
|  | *Pol high* | -100 (-100, -100) | -100 (-100, -100) | 0 |
|  | *Ref low* | -100 (-100, -100) | -100 (-100, -100) | 0 |
|  | *Ref mid* | -100 (-100, -100) | -100 (-100, -100) | 0 |
| **Motang** | *Ref high* | -100 (-100, -100) | -100 (-100, -100) | 0 |
|  | *Pol low* | 10 (-14, 31) | 0, (-10, 0) | 10 |
|  | *Pol mid* | -10 (-26, 6) | 0 (-10, 0) | 10 |
|  | *Pol high* | -100 (-100, -100) | -100 (-100, -100) | 0 |


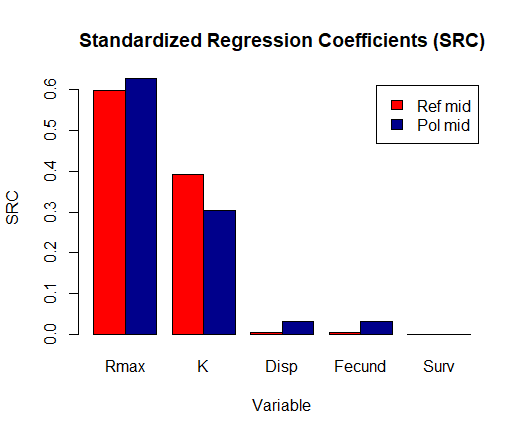


**Figure S4.1: NPM sensitivity analysis results**. Normalised standardized regression coefficients for each term in a saturated model for the response variable Expected Minimum Abundance according to our *Ref-Mid* (red) and *Pol-Mid* (blue) plausible future climate scenarios (EMA ~ Rmax + Carrying Capacity + Dispersal + Fecundity + Survival). Predictor variables are Rmax, carrying capacity (*K*), dispersal (Disp), fecundity (Fecund) and survival (Surv).

We did a further sensitivity analysis to test the influence of the reserves multiplier (1.9), which was applied only to areas within large island reserves during the calculation of K in the NPM. This multiplier was used to account for the benefit of being in a large island reserve, estimated by Purwandana et al (2015). We tested the impact of not using this multiplier on the small islands comparing the difference in EMA with and without the multiplier. The results show that our decision not to apply the multiplier to the smaller islands of Kode and Motang did not have a significant impact on the estimates of EMA in 2050 (Table S4.2).

**Table S4.2: Results of a sensitivity analysis on the impact of the reserves multiplier** on small island populations. This multiplier was only applied to populations within large reserves in the results presented in the main paper.

| **Small island** | **Pol Mid** | | **Ref Mid** | |
| --- | --- | --- | --- | --- |
|  | **EMA no multiplier** | **EMA with multiplier** | **EMA no multiplier** | **EMA with multiplier** |
| *Kode* | 7 | 9 | 0 | 0 |
| *Motang* | 9 | 12 | 0 | 0 |
